# Recent advances and opportunities related to the use of bee products in food processing

**DOI:** 10.1002/fsn3.3411

**Published:** 2023-05-15

**Authors:** Marek Kieliszek, Kamil Piwowarek, Anna M. Kot, Marta Wojtczuk, Marek Roszko, Marcin Bryła, Anka Trajkovska Petkoska

**Affiliations:** ^1^ Department of Food Biotechnology and Microbiology, Institute of Food Sciences Warsaw University of Life Sciences—SGGW Warsaw Poland; ^2^ Department of Food Safety and Chemical Analysis Prof. Wacław Dąbrowski Institute of Agricultural and Food Biotechnology—State Research Institute Warsaw Poland; ^3^ Faculty of Technology and Technical Social Sciences St. Kliment Ohridski University‐Bitola Veles North Macedonia

**Keywords:** apitherapy, bee bread, bee pollen, bee products, honey

## Abstract

Nowadays, natural foods that can provide positive health effects are gaining more and more popularity. Bees and the products they produce are our common natural heritage that should be developed. In the article, we presented the characteristics of bee products and their use in industry. We described the development and importance of beekeeping in the modern world. Due to their high nutritional value and therapeutic properties, bee products are of great interest and their consumption is constantly growing. The basis for the use of bee products in human nutrition is their properties and unique chemical composition. The conducted research and opinions confirm the beneficial effect of bee products on health. The current consumer awareness of the positive impact of food having a pro‐health effect on health and well‐being affects the increase in interest and demand for this type of food among various social groups. Enriching the daily diet with bee products may support the functioning of the organism. New technologies have appeared on the market to improve the process of obtaining bee products. The use of bee products plays a large role in many industries; moreover, the consumption of bee products and promotion of their medicinal properties are very important in shaping proper eating habits.

## Graphical abstract

The wide nutritional and therapeutic properties of bee products increase the resistance of organisms. Honey and bee products are bactericidal, antiseptic, regenerating, and nourishing. The basis for the comprehensive use of bee products in human nutrition is its diverse chemical composition.

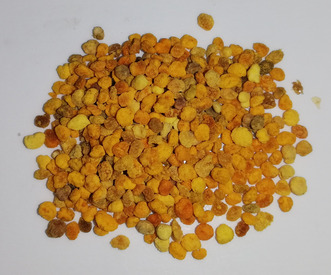

## 1 INTRODUCTION

One of the oldest examples of the existence of beekeeping is the murals from the Araña Cave in Spain, which show people collecting honey from the nest of untamed bees. In ancient times, honey was used as a medicine and also to embalm corpses. During Greek feasts, it was an indispensable part of the menu. However, the greatest boom in beekeeping took place in ancient Rome, where hives made of straw and clay were found, dating back to 900 B.C. (Kritsky, 2017). The Egyptians placed a vessel filled with a “golden drink” in the tombs of the pharaohs, which showed that it was indeed considered something special. In the beginning, people discovered that honey collected by bees can be found in the hollows of trees. They climbed these trees, taking the honey from the bees—the result of their hard work. Then, they started to breed bees, building their nests in the hollows of old trees, the so‐called beehives. Shortly thereafter, they noticed that you can only relocate the part of the tree to the field where the hollow is located, and then you will not have to climb to ensure you have constant access to this valuable product. Over time, people, thinking creatively and innovatively, came up with the idea of resettling them to today's hives.

The precursor of the scientific approach to beekeeping was Aristotle (384–322 B.C.), who studied the vital activity of bees, and their diseases and pests. Hippocrates (460–370 BCE) focused on the nutritional value and health‐promoting properties of bee products. In turn, Avicenna (980–1037 CE), a Persian scholar, studied the development of beekeeping and bee breeding. Ancient Egypt is also considered the beginning of beekeeping (Kritsky, 2015). It is believed that the first bee flew from the horn of the sacred bull—the god Apis. Hence, the Latin name of the bee is *Apis mellifera*. Lower Egypt had extensive arable land which made it the main center for beekeeping in antiquity, which is why the bee has become a symbol of this country. This fact is evidenced by documents from Egyptian beekeepers from 2400 BCE. Already in antiquity, man became convinced of the valuable taste and healing qualities of honey. Bee products and honey belonged to the group of noble products bringing considerable profits. Honey was used as the primary sweetener used to preserve products. On the basis of the obtained honey, wax, propolis, and bee bread, cosmetics, lotions, and medicines recommended for both humans and animals were prepared. The bee has grown to be something of a miracle insect (Lozo et al., 2015). In many cultures, for thousands of years, the product of bees' labor has been considered the food of the gods and used in ritual ceremonies. Honey was a sign of power, splendor, or luxury. In ancient Egypt, only Pharaoh and his dignitaries could eat honey. The beneficial properties of honey were appreciated not only in Egypt but also in ancient Greece; the Greeks believed that honey was a source of not just strength but also wisdom, which is why they sacrificed it to the gods (Qamar & Rehman, 2020). Among others, athletes ate it when preparing for competitions in the Olympics in order to improve their physical condition. History shows that the discoverer of the healing properties of honey was the father of contemporary medicine—Hippocrates. In ancient Rome, when welcoming the New Year, people drank honey in white vessels. Christians gave new followers of their religion milk and honey. On the other hand, Muhammad recommended that the followers of his faith drink honey instead of wine. No wonder then that in ancient cultures such as Greece, Egypt, and the Far East, honey was generally considered as medicine for both body and soul (Eteraf‐Oskouei & Najafi, 2013).

In the article, we present the importance and development of beekeeping. This is an obligation resulting from both the needs of life and the importance of the work of bees in nature and the economy. In addition, in the article, we discussed the characteristics of the most important bee products in general food technology and in other industrial branches. We believe that the article will be of great importance for the development and use of new technological solutions for bee products.

## 2 CHARACTERISTICS OF BEEKEEPING

For many years, the world has attached great importance to national traditions and customs. In many literary works, not only in prose, but also in verse, various traditional motifs were raised, examples of which are hunting, mushroom picking, fishing, brewing coffee, or beekeeping of the time. Descriptions of old traditions, preserved in works of literature, are sources of information for the emergence of modern customs. Old Polish beekeeping is a good example of this.

Forest beekeeping is a traditional form of forest use. Until the nineteenth century, it was the art of bee breeding, consisting of rearing bee colonies in special trees called beehives. Beehives were produced by honey hunters in remote parts of the forest. Such a location of former hives made the honeybee the dominant pollinator and a very important element of the natural organic whole of the forest, that is, the complex of all animal, plant, and microbiological organisms, called biocenosis (Dzierzanowski et al., 2009). Pine has become the most frequently chosen tree for keeping bees. There are many reasons, the most important being the abundance of this type of tree in Polish forests (at the end of the 20th century, pines covered about 70% of the stand area—a forest‐forming species), a large range of tolerance to environmental conditions, and the fact that the cutting of beehives did not reduce the viability of the trees (Sultanova et al., 2019).

In Eastern and Central Europe, the first form of beekeeping was log beekeeping – a profession inherited from generation to generation, involving the use of the natural environment. Log beekeeping played an economic, legal, and political role in the Polish lands for about a thousand years. In the years 2006–2008, the nongovernmental organization World Wide Fund for Nature (WWF) implemented a project aimed at restitution of forest beekeeping in Polish forests. The project used the practices of Ural honey hunters in order to renew the old traditions in the “Świętokrzyska Forest” and “Nadpilicka Forest” (Poland). An important aspect of the project was not only the revival of the customs mentioned in the literature but also the local restoration of the natural species structure of pollinating insects—native honeybee breeds and the protection of biodiversity (Śliwka & Staniszewski, 2014).

The efficiency of bee rearing in forest conditions, as in the case of log beekeeping, was higher than in modern apiaries. This is related to the greater amount of food in the vicinity of the forest and the fact that the bees flying out of the trees had a greater flight range than the bees living in hives placed on the ground (Korbel, 2012). Log beekeeping was less time‐consuming due to the natural living conditions of the bees. Compared to modern beekeeping, the beekeeper's attention was needed only twice a year—to clean the beehives in spring and to collect honey in the fall. In the case of modern beekeeping, year‐round inspection is needed.

Beekeeping honey deserves special attention—the fruit of the work of the honeybee keeper and the bees, which was and is a highly valued commodity. In the past, honey was such a valuable raw material that it was used as a form of cash, and honey hunters were always venerated and highly respected. Nowadays, honey is rare on the world market. The largest producers are honey hunters from Bashkiria (Ural, Russia), and their product is included among the most expensive honey in the world (Khisamov et al., 2019).

The extinction of the honeybee is associated with the replacement of log beekeeping by the apiary since the mid‐18th century, administrative prohibitions in the field of log beekeeping (Śliwka & Staniszewski, 2014), and the presence of bee parasites, including Varroa destructor, which comes from Asia. The above‐mentioned decline of log beekeeping, as well as the extinction of wild honeybees, has had a negative impact on the natural ecosystem of European forests, as beekeeping did not completely replace the tasks of wild bees. Over the years, people have found that they owe the bee colony much more than just the opportunity to obtain honey and their other products.

## 3 THE ROLE OF HONEYBEES IN FOOD PROCESSING OR FOOD INDUSTRY

A bee is an insect of great importance to humans. *Apis mellifera* is a strategic species in plant pollination and thus increases the productivity of both agriculture and horticulture. Of 100 crops of plants supplying 90% of the world's food, 70 of these plants are pollinated by bees. On the other hand, the economic income resulting from pollination significantly exceeds the value of the honey produced (Burgett, 2011;Fei et al., 2021). The importance of beekeeping results from, on the one hand, the nutritional and therapeutic value of bee products, and on the other hand—the pollination process itself. By collecting pollen from flowers and transferring it to others, the yields of fruit and seeds of insect pollen plants increase, on average, by 30%. This deliberate action of bees makes them a very important link in the ecological balance and so, being dedicated to beekeeping will not only benefit the environment but also the development of the food industry. Beekeeping has immeasurable benefits affecting many areas of the economy and public health.

The honeybee, *Apis mellifera*, is divided into species from which at least 25 geographical subspecies and races can be distinguished. In Europe, the most popular breeds used for breeding are Central European, Caucasian, and Ukrainian. In 2019, the largest consumers of honey were Germany (69,000 tons), France (52,000 tons), and the United Kingdom (45,000 tons), which accounts for 38% of consumption in the entire EU (Popescu et al., 2021). Honey consumption in Poland is generally lower than its production; therefore, 3000 to 5000 tons of honey are imported annually. The average bee yield depends on the harvest and ranges from 10 to 20 kg of honey per year. The average Pole consumes from 0.3 to 0.4 kg of honey per year and to produce 1 kg of honey, bees must visit 5.7 million flowers (Mubin et al., 2022). Usually, one bee can visit from 50 to 1000 flowers in 1 year (Sultanova et al., 2019). A necessary condition for high efficiency is to choose the right place to set up the hives and to have healthy and strong colonies of 50,000 to 100,000 bees (Grüter, 2020; Hayatu Ibrahim et al., 2021). The honeybee is a pollinator for about 70% of plant species, including wild‐growing forest flora, for which the mediation of insects is necessary in the reproduction process. This necessity was conditioned by the interaction between bees and flowering plants, created in the process of interdependent species evolution (so‐called co‐evolution). As a result of the above‐described linkage, the local disappearance of one pollinator species may result in the disappearance of the population of several plant species. An appropriate warning may be the statement that “when the bee disappears from the face of the Earth, man has only four years to live”. Since there will be no bees, there will also be no pollination. So, there will be no plants, then animals, and finally, it will be people's turn (Foer, 2019).

With the development of civilization, the number of threats to honeybees increases, becoming more and more susceptible to environmental changes (Flores et al., 2019) and diseases caused by bacteria, viruses (Chen et al., 2021), and pesticides (Yordanova et al., 2022). In order to ensure that honeybees are still one of the pillars of the ecosystem, man builds apiaries located in city centers, adapted to the current requirements for proper hygiene of obtained bee products. This is to protect apiaries from pesticide poisoning (Kaila et al., 2022). In addition, for the sake of these insects, mankind maintains unused field separation strips so that the bees can benefit from the plants growing there.

Bees are insects of great importance for nature and man. Apart from honey, their products have been a valuable raw material since the time when humans first learned to benefit from the wealth of the bee community. So far, we use honey, propolis, and bee pollen in our everyday life (Fujikawa et al., 2018), for example, for the needs of today's demanding food, pharmaceutical, and cosmetic industries. In connection with the above, it can be agreed that the hive is an exemplary model of the most efficient and longest running bee‐led factory, which for millions of years has been continuously providing valuable and necessary products with a very large application in food technology.

## 4 CHARACTERISTICS OF SELECTED BEE PRODUCTS

### 4.1 Characteristics of the honeycomb

Around 36 B.C.E., Marcus Terentius Varro, in his book on agriculture, wrote about the hexagonal form of a bee's honeycomb. There were two competing theories for the hexagonal structure. One theory was that the hexagons were a better fit for a six‐footed bee. Another theory, supported by mathematicians at the time, was that the structure was explained by the isoperimetric property of the hexagonal honeycomb (Sajjad & Lu, 2021).

**FIGURE 1:**
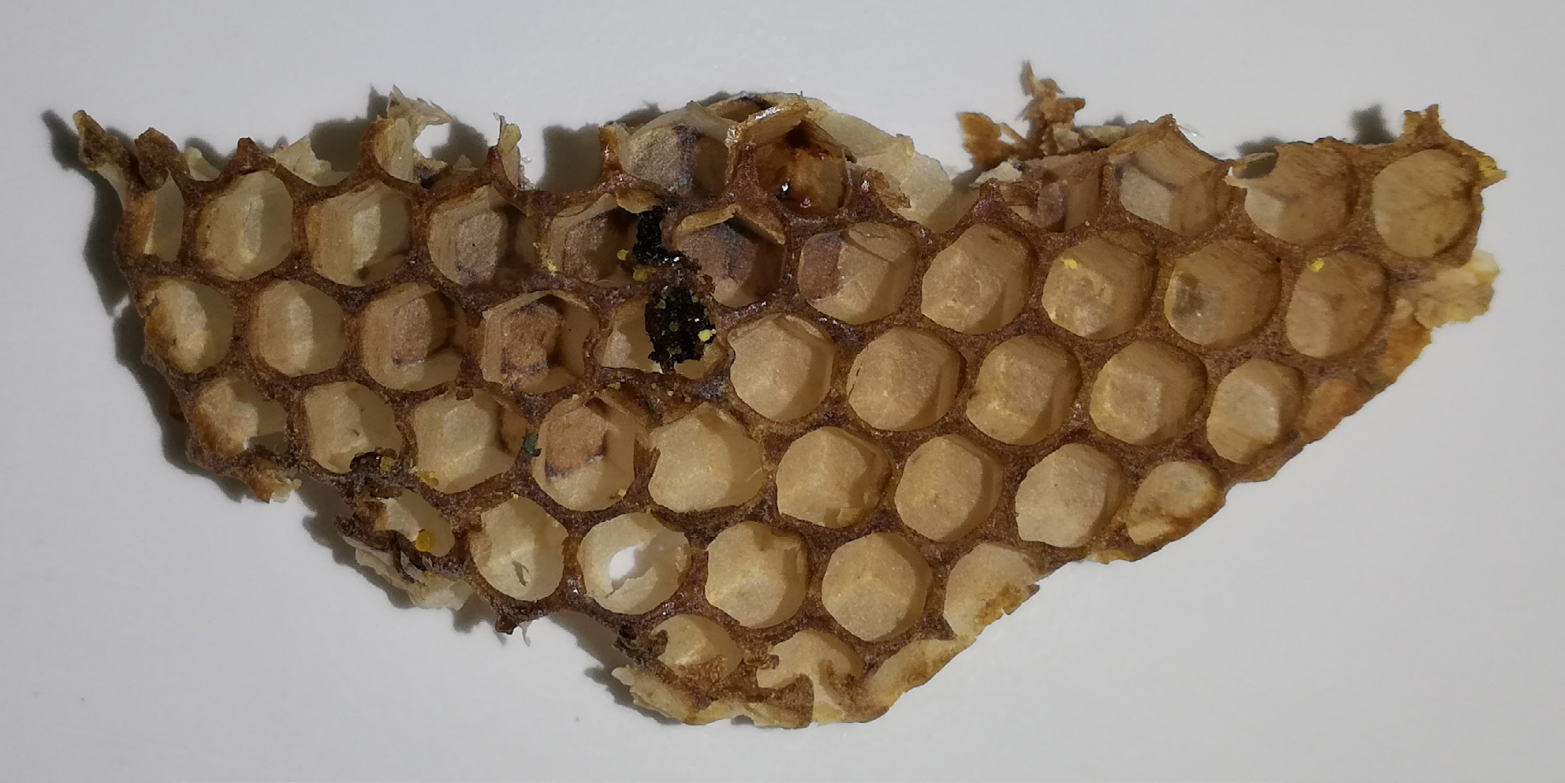
The structure of the honeycomb.

It is now known that the honeycomb (Figure 1) is a structure composed of hexagonal cells, which are built by worker bees from the wax secreted by the wax glands. The inclination of the walls in relation to each other is very refined because it is to ensure the minimum use of building materials. The honeycomb has regular hexagonal patterns on both sides, and it is known that the cell bottom is made of three equal diamonds. By creating such a perfect structure, the bees try to use the minimum amount of wax per cell (Dutta, 2021).

Honeycombs are used by bees to store supplies and rear their brood (Oeder & Schwabe, 2021). The honeycomb cells are filled by the bees one by one from top to bottom. In the comb, the bees store the nectar they get from the flowers. They then convert this nectar into honey using enzymes contained in their saliva. After filling the cell with honey, the bees close it tightly with a thin layer of wax. This process is called sealing. During the winter, when bees have nowhere to obtain pollen or nectar, they gnaw at the capsule and begin to eat the supplies they have accumulated over the summer (Breed et al., 1995). In other cells, bees store pollen, which they also collect from flowers. Then, after adding a little honey and enzymes present in their saliva, a bee bread is formed. By mixing bee bread with honey and royal jelly, bees produce a mixture that is fed to the brood, that is, young larvae. In the remaining cells, the queen bee lays one egg in each, which forms her brood. The larvae are fed by the bees and mature in their cells. After some time, when the larvae reach the correct size, the bees close the cell, and the larva turns into a bee. After this transformation, the young bee begins to bite the hole in the cap with its rumen and leaves the cell. The color of the patch varies from white to brown to almost black. This is due to the aging process (Breed et al., 1995).

### 4.2 Honey

Honey is the most popular bee product, it is a natural, sweet substance produced by the honeybee. The basis for the production of honey is flower nectar and honeydew (Baloš et al., 2020), these are processed in the bee's organism and mixed with its secretions, and then dehydrated and left in combs for maturation (Çelik & Aşgun, 2020). Honey varieties differ widely from each other, these differences are primarily influenced by the origin of the nectar from which the honey is to be made, and the climate, place of origin or soil are also important (Schellenberg et al., 2010). Honey as defined in Council Regulation (EC) No 1234/2007 of 22 October 2007 (Regulation 2007/1234) means a naturally sweet substance produced by *Apis mellifera* bees from the nectar of plants or the secretions of living parts of plants, or the secretions of insects sucking up live parts of plants, collected by bees, processed by combining specific substances from bees, folded, dehydrated, collected, and left in the honeycombs for maturation. Honey standards are laid down in the European Honey Directive and in the Codex Alimentarius standard (CXS 12‐1981 1, 2019; Regulation 2007/1234, 2007). Honey meets the requirements for commercial quality if: it does not have an odor and taste unusual for a given variety of honey, and the acidity of the honey has not been artificially changed. In addition, the natural enzymes found in honey have not been partially or completely destroyed by heating. Pollen or any other specific honey component has not been removed from it, except in filtered honey, unless it was unavoidable in the process of removing extraneous organic or inorganic matter (Regulation 2001/110/EC, 2001). Honey is always in high demand, mainly due to its sweet taste and rich nutritional value. For thousands of years, it has been used for nutritional purposes as a sweetener and therapeutic agent. It was to ancient civilizations what aspirin is to today's medicine. Organoleptic properties, which include aroma, taste, color, and texture, determine the good quality of honey. The parameters of individual varieties are varied and depend on the type of raw material from which the product was made. The geographic region, climate, species of bees that produce honey, and the conditions in which the honey is processed, packaged, and stored also have a great influence (Machado De‐Melo et al., 2018).

Honey is classified as healthy food, the main components of which are carbohydrates and water (below 20%) (Yan et al., 2022). Dry matter of honey consists of carbohydrates (Kolayli & Keskin, 2020), responsible for the physicochemical properties of this product, which include viscosity, hygroscopicity, and granulation. The sugar contained in honey is mainly fructose and glucose, and to a lesser extent sucrose, which are easily absorbed by the body, and their proportionality depends on the type and variety of honey (Chambó & de Toledo, 2020). The chemical composition of various honey types is presented in Table 1.

**TABLE 1:** Chemical parameters and nutritional ingredients of various honey types (Alvarez‐Suarez et al., 2018; Morroni et al., 2018; Tomczyk et al., 2019).

Parameters	Type of honey							
	Multifloral	Multifloral	African	Manuka	Tilia	Forest	Rape	Acacia
Moisture (%)	18.65	16.74	12.26	10.22	17.76	17.40	17.86	17.73
Free acidity (mEq/kg)	37.0	32.65	23.01	21.63	34.20	33.60	18.60	25.60
pH	3.57	4.76	4.83	4.51	3.81	4.16	3.88	3.79
Color intensity (mAU)	699	Nd	Nd	Nd	820	1098	427	422
Fructose (%)	39.23	Nd	Nd	Nd	36.89	34.67	36.35	37.64
Glucose (%)	31.82	Nd	Nd	Nd	28.43	29.10	32.92	31.75
Sucrose (%)	4.40	Nd	Nd	Nd	6.12	6.29	5.06	3.53
DPPH (% inhibition)	36.12	Nd	Nd	Nd	40.71	60.52	21.21	24.80
FRAP (mmol TE/kg)	1.32	Nd	Nd	Nd	0.95	60.52	0.68	1.59
ACW (mmol AA/kg)	16.10	Nd	Nd	Nd	12.62	19.09	9.93	15.77
ACL (mmol Trolox/kg)	2.19	Nd	Nd	Nd	1.48	2.34	0.67	1.73
TPC (g GAE/kg)	0.46	Nd	Nd	Nd	0.38	0.60	0.25	0.47
Flavonoids (mg QE/kg)	4.96	Nd	Nd	Nd	4.99	6.68	3.24	3.15
Total carotenoid content (TCC) (mg βcarotE/kg of honey)	Nd	4.78	5.21	4.63	Nd	Nd	Nd	Nd
Vitamin C content (VitC) (mg/100 g of honey)	Nd	4.55	2.73	2.84	Nd	Nd	Nd	Nd
Folic acid content (mg folic acid/100 g of honey)	Nd	8.34	5.46	0.61	Nd	Nd	Nd	Nd
Total free amino acid content (mg LE/100 g of honey)	Nd	99.15	61.19	185.49	Nd	Nd	Nd	Nd
Total protein content (mg BSA/g of honey)	Nd	1.81	1.35	2.62	Nd	Nd	Nd	Nd

Abbreviations: ACL, antioxidant capacity of lipid; ACW, antioxidant capacity of water soluble; DPPH, 2,2‐diphenyl‐1‐picrylhydrazyl; FRAP, ferric reducing antioxidant power; Nd, no data; TPC, total phenolic content.

The amount of water in honey is extremely important as it determines the taste, quality, and properties of the product (Serra Bonvehí & Ventura Coll, 2003). In addition, water, which should be between 17% and 20%, has an influence on the color, crystallization, viscosity, taste, and density of the honey. The low water content is determined by external factors, namely, the humidity and temperature of the environment during the processing of nectar by bees, and the conditions that prevail during human harvesting. Its excess can lead to the fermentation of honey and the development of *Candida* yeast, spore bacteria of the genus *Clostridium* and *Bacillus*, which are resistant to dehydration and high sugar concentration (Almasaudi, 2021).

Honey, in its composition, has a relatively small amount of proteins (from 0.2% to 1.6%), which are amino acids derived from bees, pollen, and nectar. Among the amino acids, the most common is proline, the others are, inter alia, glutamic and aspartic acid, glutamine, lysine, and tyrosine. Proline comes mainly from the salivary secretions of the honeybee and constitutes about 50%–85% of the amino acids in honey. Proline is a measure of the ripeness of honey, and its content in honey should exceed 200 mg/kg. Values below 180 mg/kg mean that the honey is probably adulterated with added sugar (Dessie, 2021; Gorjanović et al., 2013).

Most of the proteins in honey are enzymes such as invertase, amylase, and glucose oxidase, which are chemically essential components of honey. An example may be the above‐mentioned enzyme—glucose oxidase, which produces hydrogen peroxide, having a bacteriostatic effect. These enzymes usually come from the salivary glands of bees and to a lesser extent from the collected pollen and nectar. Bees enrich the nectar or drop enzymes already during their flight back to the hive, then the honey is treated with enzymes in the combs during the further maturation process (Da Silva et al., 2016; Flanjak et al., 2016).

The distinctive ingredient of each variety of honey is organic acids, which are responsible for its taste, aroma, and texture (Aparna & Rajalakshmi, 2009) and for the proper acidity of the honey—stopping the fermentation process. Organic acids in honey are present in small amounts; they constitute <0.5% of honey components. The predominant organic acid in honey is gluconic acid (Kawashima et al., 2019; Valverde et al., 2022), others include succinic, acetic, malic, and citric acids (Seraglio et al., 2021). The low acidity of honey in the range from pH 3.2 to pH 4.5 contributes to the antibacterial effect of gluconic acid (Almasaudi, 2021).

Honey contains small amounts of minerals such as iron, magnesium, potassium, calcium, and sodium, with the highest concentrations of potassium, sodium, calcium, and magnesium (Akbulut et al., 2009; Alves et al., 2013). Honey is not a vitamin‐rich product (Ragab et al., 2020). It contains vitamins A, E, and K as well as B vitamins (Meo et al., 2017) such as vitamin B1, vitamin B2, and vitamin B6, which support the immune system. Folic, nicotinic, and pantothenic acids are also present, which improve the functioning of the circulatory and nervous systems (Rosiak & Jaworska, 2019; Zaid et al., 2021).

#### 4.2.1 Classification of honey

The expanding global market for honey has stepped up efforts to authorize and characterize honey as they play an important role for both consumers and producers. Depending on the raw material from which it was made, honeydew and nectar honey are distinguished (Sanz et al., 2005). Sweet fall is the term for the droplets formed on the needles of spruce, larch, and fir as well as on the leaves of, among others, linden or oak. Honeydew consists of plant juices that come out of plant cells damaged by aphids, honeydews, or scions. Honeydew juices are mainly sugars and minerals derived from plant gland secretions (Mežnarić et al., 2022) that attract pollinating insects, including bees.

Two types of honeydew honey are most common in Europe, that is, honeydew honey and coniferous honeydew. Coniferous honeydew honey are distinguished by a very dark color. Honeydew honey have an unusual taste: from conifers—mild, slightly resinous, and from deciduous trees—slightly spicy and tart. Moreover, honeydew honey have various physical and chemical properties. In recent years, there has been an increased interest in consuming this type of honey as a functional product. Their valuable biological properties have now been appreciated because they have a lower content of simple sugars, higher acidity, more protein, and minerals such as iron and magnesium—in relation to nectar honey (Flanjak et al., 2016; Seraglio et al., 2021). Coniferous honeydew supports the treatment of heart and rheumatic diseases, and has an antiseptic and anti‐inflammatory effect; therefore, coniferous honeydew honey is used in the treatment of the respiratory tract. It consists of vitamin C, vitamin K, vitamin PP, B vitamins, and essential oils, which is the reason that it is also consumed in states of reduced immunity. Leafy fall is used in diseases of the biliary tract, urinary system, and joint pains, and has anti‐inflammatory and disinfecting properties (Mroczek & Mroczek, 2020).

Examples of nectar honey include multiflorous and single‐flowered honey, rapeseed, raspberry, acacia, buckwheat, and heather honey, which differ in their health‐promoting properties and composition influenced by the richness and diversity of bee or honey flora and the type of plants from which the nectar is obtained (Chambó & de Toledo, 2020).

Buckwheat honey has a diuretic, anti‐inflammatory, and disinfecting effect. It is used in the treatment of heart diseases, it helps to lower blood cholesterol and high blood pressure, and helps in the treatment of the digestive system, including gastric and duodenal ulcers (Hołderna‐Kędzia et al., 2012). Due to its high iron content, it is used in the treatment of anemia (Farooq et al., 2020). In addition, it includes macronutrients such as phosphorus and magnesium as well as vitamins. It is not as sweet as traditional honey. It is amber in color with a slight red tinge (Chirsanova et al., 2021).

Acacia honey is almost transparent in color, has a floral aroma, and has a sweet, delicate flavor. It stays liquid longer and crystallizes much more slowly than traditional honey, which is due to its higher fructose content. It contains small amounts of vitamins and minerals such as vitamin C and magnesium. On the other hand, it is distinguished by a high content of flavonoids with strong antioxidant properties (Hołderna‐Kędzia et al., 2012). Additionally, this honey helps maintain a moist environment while providing a protective barrier that can aid in wound healing (Andreu et al., 2015).

Linden honey, due to the content of hesperidin (Zhao et al., 2015), essential oils, tannins, ascorbic acid, carotene, and organic acids (Consonni & Cagliani, 2015), is considered to be the best remedy for colds because it has warming and antiseptic properties (Bertoncelj et al., 2007), but also has a calming effect and lowers blood pressure. It has high antibacterial activity; therefore, it is used in diseases of the upper respiratory tract.

Rapeseed honey is used in diseases of atherosclerosis and the biliary tract, and in treating inflammation of the upper respiratory tract and urinary tract. It consists of vitamin K, vitamin C, B vitamins, mineral salts, essential oils, flavonoids, tannins, and amino acids (Chirsanova et al., 2021) which support the liver by increasing its detoxification capacity (Hołderna‐Kędzia & Kędzia, 2021). Rapeseed honey contains essential oils, tannins, and flavonoids (mainly quercetin, kaempferol, and apigenin). This type of honey has the ability to nourish and regenerate an exhausted body (Sidor et al., 2022).

Raspberry honey helps to replenish iron deficiency in the body. It is used in colds, upper respiratory tract diseases, and atherosclerosis. Honey consists of minerals and vitamins as well as compounds such as potassium, magnesium, calcium, iron, and manganese (Hołderna‐Kędzia & Kędzia, 2021).

Heather honey has healing properties in diseases of the urinary tract, the mucosa of the mouth and throat, inflammation of the palatine tonsils, intestinal ailments, and diarrhea (Moise et al., 2013). It contains vitamins C, A, B2, B6, PP, and iron (Ioĭrish, 2001).

#### 4.2.2 Honey extraction and storage

Recently, new beekeeping technologies have appeared on the market, enabling more efficient obtaining of honeybee products. These processes consist of extracting and de‐aerating the honey, clarifying and sterilizing the wax, and extruding wax foundation (template with embossed shapes of honeycomb cells). Electric vacuum cleaners are used for this purpose (Archibong, 2021). For uncapping the combs with ripe honey, fork uncappers or electronically heated knives, planes, or hot air are used. A honey extractor (centrifuge) is used to centrifuge honey from honeycombs (Kadri et al., 2017). Innovative methods of selecting honey, thanks to which the honey flows straight into the jar, require the use of specially constructed frames or whole hives. Honey picking is done from closed hives. After the honey extraction process, it should be strained through a set of sieves and clarified to get rid of impurities (Bicudo de Almeida‐Muradian et al., 2020). In order to obtain a product with a creamy consistency, the honey is mixed when the first crystals appear. Honey can undergo a decrystallization process by heating it to a temperature of 40°C, which deteriorates its quality (Baglio, 2018). The water used in the cleaning of sieves, uncappers, honey extractors, and for rinsing a large number of plugs is used to feed the swarms or undergoes fermentation, which results in the production of mead (Pędziwiatr & Zawadzki, 2017).

Natural honey, if properly stored, retains its characteristic quality for a longer period of time (Singh & Singh, 2018). The quality of the honey in the storage process is influenced by such factors as the technological parameters of the room, humidity, temperature, and type of packaging. Too high humidity leads to the development of fungi, and their development causes the fermentation of sugars (Eshete & Eshete, 2019). To prevent honey fermentation, the appropriate storage temperatures are between +5°C and +10°C. Honey packaging is also very important. It is recommended to use barrels made of poplar, linden, alder, aspen, or stainless steel tanks as well as glass containers (Tihonow et al., 2017).

Literature data confirm that honey is one of the most valuable pro‐health products and should be included in the daily diet (Samarghandian et al., 2017). Consumed directly or as an addition to dietary supplements and other food, it has a positive effect on the human body. Honey is also a substance used in medicine and cosmetology. The use of honey in medicine is determined primarily by its high sugar concentration as well as its organic acids, vitamins, and microelements important for health (Meo et al., 2017). In addition, it has anti‐inflammatory and bactericidal properties, comparable to treatment with antibiotics. On the other hand, the polyphenols contained in honey have a beneficial antiradical effect (Otmani et al., 2021). Moreover, thanks to its valuable biological properties, honey is a moisturizing ingredient in various preparations (El‐Senduny et al., 2021).

#### 4.2.3 Properties of honey

Honey has many benefits for human health and has been used in traditional and complementary therapies for many years. The healing properties of honey are also largely due to the polyphenolic compounds (e.g., phenolic acids: chlorogenic, caffeic, ellagic, ferulic, gallic, p‐coumaric), mainly flavonoids (e.g., catechin, chrysin, kaempferol, luteolin, naringenin, pinocembrin, rutin, and quercetin) (Tanleque‐Alberto et al., 2020) transferred by bees from nectar to honey. Almasaudi (2021) in his article on the antibacterial effects of honey pointed out that the highest concentration of polyphenolic compounds comes from nectar collected at the beginning of June, and the main phenols of these compounds are flavonoids and phenolic acids. On the other hand, honey obtained from nectar collected in May, July, or August show a lower concentration of these compounds, and their content in the finished product is influenced by the geographical location, type of nectar, and season of the year. Flavonoids and phenolic acids are scavengers of toxic free radicals. They prevent oxidative damage to cells that lead to aging, cancer, metabolic disorders, and cardiovascular dysfunction (Almasaudi, 2021; Ferreira Da Cruz et al., 2019).

Honey has strong anti‐inflammatory, antiviral, and antibacterial properties. There is now evidence that honey plays an essential role in the prevention of cancer. It reduces the viability of cancer cells, regardless of time and concentration. The risk of developing cancer includes, among others, chronic gastric ulcers, one of the most common diseases affecting people. This risk factor is caused by the bacterium *Helicobacter pylori*, which attacks the lining of the stomach, making it difficult for wounds to heal. Research shows that one of the bee products, Manuka honey, in combination with other compounds supporting treatment has shown antiulcer activity. It reduces ulcerations and completely protects against mucus changes, which has a protective function, preventing tissue inflammation (Almasaudi, 2021; Ferreira Da Cruz et al., 2019; Othman, 2012).

Honey shows antibacterial activity against many bacteria in a variety of environments. The natural ingredients of honey have a wide range of effects on microorganisms. Godlewska and Świsłocka (2015) examined the effect of 11 different honey varieties on the degree of inhibition of the growth of bacteria such as *Staphylococcus aureus* and *Escherichia coli*. On the basis of the obtained results, it was found that a 50% concentration of honey effectively inhibits the growth of bacteria. Dark honey (buckwheat, heather, and forest honeydew), eliminating the growth of *Escherichia coli*, had the strongest effect. In the case of *Staphylococcus aureus*, forest, heather, and buckwheat honey showed effective growth inhibition compared to the other varieties whose growth inhibitory effect was weaker. On the other hand, the remaining varieties of honey (honeydew honey, dandelion, linden, rapeseed, acacia, and raspberry) showed less activity against the strains tested. Based on the results, it was found that the honey samples had different levels of inhibitory activity at different concentrations in relation to the tested microorganisms, with zones of inhibition increasing with increasing honey concentration. The analysis of the obtained results confirmed that all honey, both light and dark, had antimicrobial properties. It is worth emphasizing that dark honey inhibited most strongly the growth of gram‐negative bacteria in relation to gram‐positive bacteria. According to Syed Yaacob et al. (2020), the inhibitory and killing properties of *Heterotrigona itama* on pathogenic bacteria remain unresolved.

Another study by Iacopetti et al. (2020) on Manuka honey from New Zealand demonstrated that the honey is effective against hemolytic streptococci and enterococci. This honey is unique in the fact that it contains methylglyoxal (Gośliński et al., 2020), a bactericidal substance found in the nectars of the Manuka tree flowers, that is, *Leptospermum scoparium*. Honey Manuka influences, inter alia, the reduction of microorganisms located in the skin (Johnston et al., 2018). It has been used as an ingredient in dressings to treat burns, which accelerated the healing of skin tissue (Iacopetti et al., 2020). On the other hand, the analysis of honey samples taken from various geographic locations in Nigeria showed that with the increase in the concentration of honey, the development of *Saccharomyces*, *Candida*, *Penicillium*, and *Aspergillus fungi* is effectively inhibited. Nigerian honey can be used to make antifungal drugs, especially against candidiasis (Khan et al., 2018). Honey samples were tested for their antifungal activity and the obtained results allowed concluding that these honey had different inhibition dynamics at different concentrations in relation to the tested fungal—the inhibition zone increased with increasing honey concentration (Ferreira Da Cruz et al., 2019).

Honey is one of the oldest sweeteners consumed by humans (Abbas et al., 2021). It also has long‐lasting special properties and it is one of the few food products in the world that does not spoil easily (Schmidt, 1997). The high sugar content and low water content as well as the acidic nature and antimicrobial enzymes produced by bees are properties that help them survive for a long time (Cheepa et al., 2022). Microorganisms naturally occurring in honey in small amounts do not pose a threat to human health—they are yeasts and molds, derived from pollen, bee's digestive tract, dust, air, and flowers. On the other hand, the presence of *Clostridium botulinum* (Yap et al., 2022), which is harmful to children, causes infant botulism (Goderska, 2022)—a disease that damages the nervous system, causes respiratory failure and paralysis, and has been detected in honey. *Clostridium botulinum* is capable of producing spores that are resistant to adverse environmental conditions, so they can withstand processing and storage for long periods. The presence of an increased number of microorganisms in honey, such as *Escherichia coli*, coliform bacteria, *Penicillium*, and *Saccharomyces*, which could become a hazard to human health, may indicate secondary contamination during human processing. Due to improper storage, honey may also lose some of its health‐promoting properties (Seraglio et al., 2021; Sereia et al., 2017).

### 4.3 Bee pollen

**FIGURE 2:**
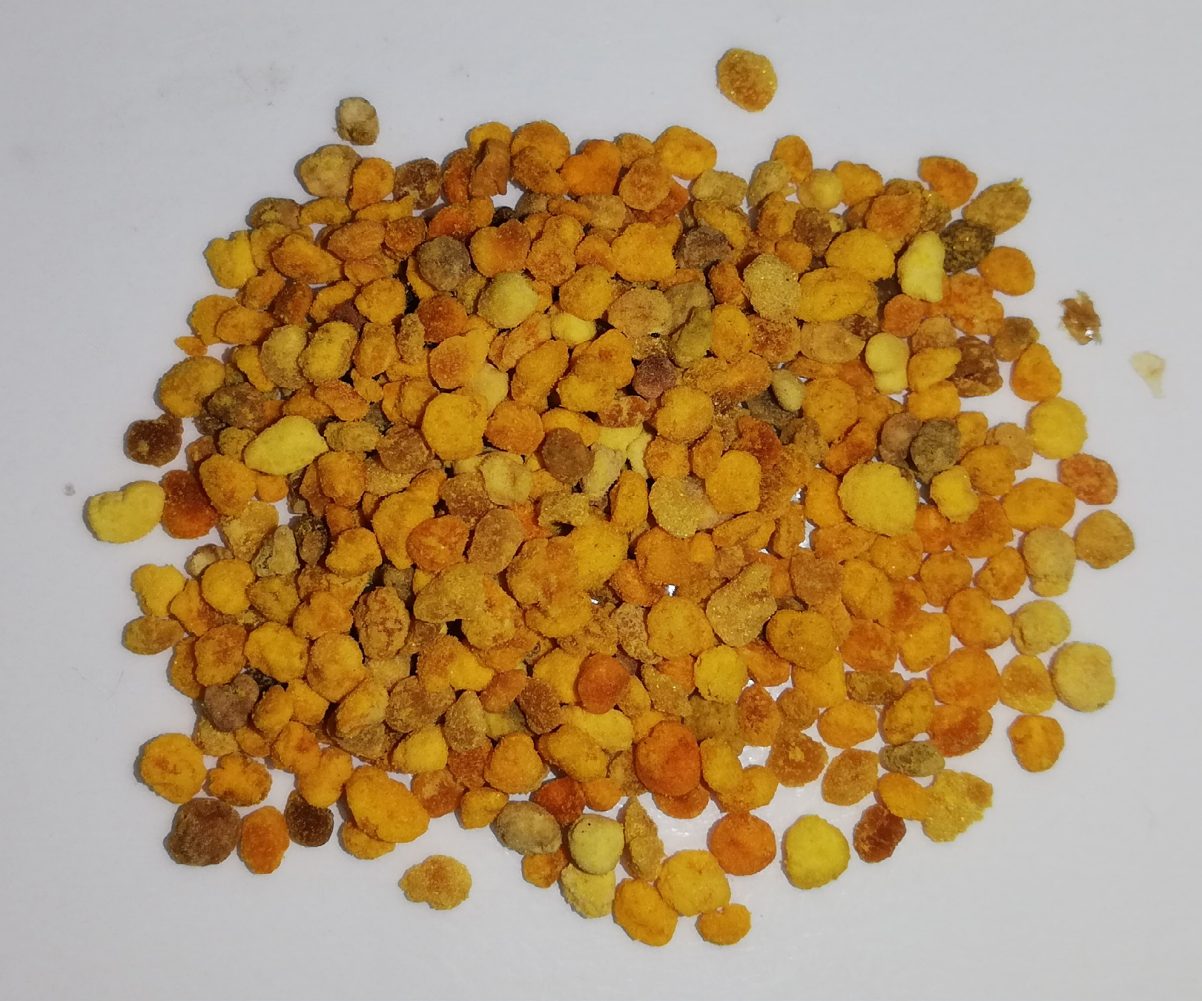
Bee pollen grains.

Bee pollen is a natural product collected and processed by honeybees. Bee pollen grains (Figure 2) are collected from plants and transferred to the hive, where they are used as raw material for the production of food for the bee colony. The bee collects pollen from the flowers with its jaws and front legs, moistening it with a little honey, saliva, or nectar. In the form of shaped balls, the “golden powder” is transported to the hive in a special basket placed on the hind legs (Komosinska‐Vassev et al., 2015). In the hive, the bees store pollen in the comb cells with a little honey to prevent spoilage and maintain the correct quality. Stored pollen, during storage, due to the heat inside the hive, is subjected to the process of lactic fermentation, where some proteins are broken down into amino acids (Di Cagno et al., 2019). After undergoing this process, pollen is referred to as bee bread (Bayram et al., 2021; Kieliszek et al., 2021).

Bee pollen is considered to be one of the most nutritious natural foods for both bees and humans (Filipiak et al., 2017). It is dark orange, light yellow, or brown in color. Sometimes, it can have a shade of green or red. Due to its valuable composition, it is consumed by humans in the form of granules as a dietary supplement and used as an additive to functional food (Khalifa et al., 2021). The impressive nutritional profile of bee pollen has been proven by numerous studies (Ares et al., 2018). Pollen granules contain over 250 different substances, mainly carbohydrates, proteins, fatty acids, vitamins, minerals, and polyphenols (Table 2). The content and quantity of the ingredients vary according to the plant species, season, and climatic zone in which the bees collected it (Peršurić & Pavelić, 2021). About 35% of bee pollen is sugar, mainly fructose and glucose. The highest percentage of compounds, about 40%, are proteins, which depends on the botanical origin of the plants and the season. Studies have shown that bee pollen obtained from pine trees contained about 7% protein, while pollen collected from date palms contained 35% protein (Ares et al., 2018; Kieliszek et al., 2018; Thakur & Nanda, 2020).

**TABLE 2:** Chemical composition of bee pollen (Campos et al., 2008; Thakur & Nanda, 2020).

Compound	Value
[%]	
Carbohydrates	13–55
Crude fiber	0.3–20
Protein	10–40
Fat	1–13
[mg/100 g]	
Potassium	400–2000
Phosphorus	80–600
Calcium	20–300
Magnesium	20–300
Zinc	3–25
Manganese	2–11
Iron	1.1–17
Cooper	1.1–17
β‐Carotene	1–20
Tocopherol	4–32
Niacin	4–11
Pyridoxine	0.2–0.7
Thiamine	0.6–1.3
Riboflavin	0.6–2
Folic acid	0.3–1
Pantothenic acid	0.5–2
Biotin	0.05–0.07
Ascorbic acid	7–56

The amino acids present in the pollen that come from the nectar are responsible for the pleasant smell of the pollen product. The dominant amino acids are glycine, aspartic acid, glutamic acid, alanine, leucine, valine, lysine, serine, and isoleucine (Taha et al., 2019). Pollen harvested in the fall has a different amino acid composition to that produced in summer and spring (DeGrandi‐Hoffman et al., 2018) because it contains a high proportion of pollen harvested from autumn flowering plants with insufficient protein content. Enzymes are also a component of pollen, which facilitate the breakdown of food substances, thanks to which these substances are more easily absorbed by the body. These include, among others, alpha‐amylase, beta‐amylase, invertase, lipase, and alkaline phytase, derived from plants and bee saliva. Alpha‐amylase and beta‐amylase break down starch and glycogen. Invertase breaks down sucrose. Lipases break down lipids and phospholipids. An important role is played by the alkaline phytase that breaks down phytic acid, thanks to which it is possible to increase the bioavailability of nutrients contained in pollen (Barrientos et al., 1994; Kurek‐Górecka et al., 2017).

Bee pollen contains 0.3% to 20% of lipids (Rzepecka‐Stojko et al., 2015). In Polish pollen, lipids contain palmitic acid, arachidic acid, and stearic acid belong to saturated fatty acids. The composition of pollen also includes valuable mono‐ and polyunsaturated fatty acids (omega 3 and omega 6), phospholipids, and phytosterols. Polyunsaturated fatty acids are essential for the proper growth and development of children and adolescents. Phospholipids present in pollen are involved in metabolic transformation; they prevent the accumulation of fat and cholesterol in the liver. This ingredient should be included in the daily diet of menopausal women (Pascoal et al., 2014).

Polyphenols are another group of bee pollen components that determine its positive impact on human health through their antioxidant activity (antioxidant, anti‐inflammatory, antibacterial) (Fatrcová‐Šramková et al., 2013; Habryka et al., 2021). These compounds also affect physicochemical properties such as color, taste, and smell. Their content varies depending on the origin of the raw material and they constitute about 3%–5% of the product of bees' labor. Due to their structure, polyphenols in pollen can be divided into flavonoids and phenolic acids (Carpes et al., 2012). During studies of the chemical composition of pollen legs, various forms and types of flavonoids were discovered, that is, quercetin and kaempferol (Pérez‐Pérez et al., 2012). Among them, the most important element is rutin (rutoside), classified as quercetin glycoside—the structure responsible for the biological properties of pollen. On the other hand, rutin in the human body contributes to the strengthening of blood vessels and acts as a natural antioxidant (Komosinska‐Vassev et al., 2015; Rzepecka‐Stojko et al., 2015).

The product of bees' work in the form of bee pollen is a rich source of vitamins, both water‐soluble and fat‐soluble. The water‐soluble vitamins are from the B and C vitamin groups, while the fat‐soluble ones include provitamin A (carotenoids and beta‐carotene), vitamin D, and vitamin E. The information contained in the scientific literature confirms that bee pollen is an unusual product containing almost the entire vitamin complex (Kurek‐Górecka et al., 2017). In addition, the valuable substances in pollen include bio‐elements, including macro‐elements (phosphorus, calcium, potassium, magnesium, and sodium) and microelements (zinc, copper, iron, manganese, silicon, and selenium) (Erdoğan et al., 2023; Komosinska‐Vassev et al., 2015).

#### 4.3.1 Industrial use of bee pollen

The properties of bee pollen translate into more and more common use in food technology. The industry uses both natural bee pollen and its extracts, but the latter are used more often (Kurek‐Górecka et al., 2020). It is also recommended to supplement the diet with pollen for athletes and the elderly due to its positive effect on metabolism and muscle work. Pollen is already widely used in Chinese clinical practice and the German Federal Office of Health has officially recognized pollen as a medicine (Salles et al., 2014).

Bee pollen is considered an excellent dietary supplement in everyday human nutrition; therefore, it comes in various forms (granules, capsules, powders, tablets, etc.). There are numerous food products containing this valuable raw material on the food market. It can be mixed with many products such as honey, butter, cottage cheese, or yogurt. Supplementation is recommended for learners who have problems with concentration or are malnourished due to the content of this raw material of a wide range of vitamins and amino acids, and thanks to its adaptogenic properties, it supports the work of the brain. Today, bee pollen is often recognized as the only perfectly complete food and probably the best food in the world (Kopczyńska et al., 2018; Kostić et al., 2020; Kurek‐Górecka et al., 2017).

The rich composition of bee pollen, and its nutritional and healing properties cause that there are more and more studies on the use of this raw material in food systems as a nutrient or to improve the properties of food products. Bee pollen has great potential for nutritional applications. Karabagias et al. (2018) described that the addition of ground bee pollen (0.5%, 1.0%, 2.5%, and 3.0%, w/v) to cow, goat, and sheep milk yogurts resulted in food products having higher antioxidant capacity and total phenol content. In addition, the taste, aroma, appearance, and cohesiveness of the yogurt products were increased. Another research group applied the addition of multiflora bee pollen to gluten‐free bread. It made that the physical and chemical properties of the loaves produced were improved. The addition of bee pollen to the dough resulted in a well‐leavened dough. What is more, there were no major issues with dough machinability or gassing capability during fermentation process. The pollen‐enriched breads, compared to the controls ample, were characterized by, among others: higher length, smoother, finer crumb grain, and desirable crust color (Conte et al., 2018). Conte et al. (2018) also found that the pollen‐fortified breads were softer and were characterized by a slower firming kinetic. The overall acceptability of breads supplemented with bee pollen between 3% and 5% was higher compared to the control (Khalifa et al., 2021). Krystyjan et al. (2015) used bee pollen (10%) as an additive to wheat flour to produce biscuits. The resulting cookies contained elevated levels of protein, sugar, ash, polyphenols, fiber, and antioxidant potential. Yerlikaya (2014) investigated the effect of adding bee pollen to fermented milk beverages. Supplementation of fermented milk products with pollen increased the viability of probiotic microorganisms and the viscosity of the beverages, importantly without affecting the sensory properties of the product. Thakur and Nanda (2019) created polyphenol‐rich vacuum‐dried milk powder using rape bee pollen. There are studies that suggest the use of bee pollen in the production of meat products as a natural substitute for antioxidants (inhibition of fat oxidation in black pudding or pork sausages) (Anjos et al., 2019; de Almeida et al., 2017).

Bee pollen can be used in the production of various types of food. However, it should be remembered about the risks associated with its use or consumption. Bee pollen can be a source of potential impurities (bacterial and fungal toxins, heavy metals, pesticides), and it can also cause allergic reactions. Poor and unhygienic conditions for the production or storage of bee pollen may favor the development of microorganisms, including pathogenic ones. The greatest risk is fresh pollen, bee pollen after drying is considered microbiologically safe (Kostić et al., 2015; Mauriello et al., 2017; Thakur & Nanda, 2020). Certainly, before using it in food production, bee pollen should be checked for the presence of toxins, heavy metals, or pathogenic microorganisms.

Due to its hepatotoxic and detoxifying effects, bee pollen extract may be helpful in cases of poisoning with drugs or organic substances. Bee pollen is also recommended for the treatment of liver diseases and in acute and chronic inflammation, initial degenerative states as well as toxic and posttraumatic lesions of this organ (Denisow & Denisow‐Pietrzyk, 2016). Pascoal et al. (2014) report that pollen can be used in combination with antibiotics and no microorganisms showing the ability to obtain resistance to bee pollen were detected. This bee product is also used in the case of hyperlipidemia and atherosclerosis. It reduces the level of lipids and cholesterol by 30%. Due to the presence of unsaturated fatty acids, phospholipids, and phytosterols, pollen has hypoglycemic properties. It can be used prophylactically in relation to heart disease and stroke. Bee pollen also has antiallergic properties and it prevents the release of histamine, which is one of the main substances responsible for nearly 390 allergic reactions, and protects mast cells against degranulation (Szabat et al., 2019). This process is attended by, inter alia, linoleic and linolenic acids (Lopes et al., 2019). Bee pollen also has antimutagenic activity (Karkar et al., 2021). The antitumor activity of pollen is probably related to its antioxidant properties, limiting the formation of reactive oxygen species (ROS) and the ability to induce apoptosis and stimulate tumor necrosis factor (TNF‐α) secretion. Pollen extract has a cytotoxic effect on leukemia cells and prostate cancer cells (Shahraki et al., 2019). It also helps in reducing the side effects of chemotherapy and radiotherapy. Pollen is also used in the treatment of diseases such as prostate enlargement or chronic nonbacterial prostatitis (Denisow & Denisow‐Pietrzyk, 2016).

Pollen supplementation has a positive effect on both human and animal organisms. It affects the regulation of protein metabolism and helps in cell regeneration in malnutrition (Denisow & Denisow‐Pietrzyk, 2016). Bee pollen supplementation brings benefits to athletes and the elderly due to the stimulation of muscle mass growth and supporting muscle work (Salles et al., 2014). The substances contained in the pollen influence the condition of the skin; therefore, it is used for care and beauty purposes. In this regard, amino acids found in pollen play an important role and they have the ability to prevent apoptosis induced by Ultraviolet radiation (Xi et al., 2018). The vitamins contained in the pollen delay the aging process of the skin and have anti‐wrinkle, regenerating, and brightening properties. They help to alleviate inflammation in seborrheic diseases and have an anti‐itching effect, for example, in the case of seborrheic dermatitis. The antiallergic effect of pollen is due to gamma‐linoleic acid (Kopczyńska et al., 2018).

The use of bee pollen in animal nutrition strengthens endurance and improves animal performance. Bee pollen is recommended especially in the diet of dogs participating in cynological sports, which are associated with high energy expenditure of the body. Supplementation with pollen improves the condition of the skin and hair; therefore, it is also recommended for exhibition animals. When used in birds, in addition to improving the condition of their bee bread, it reduces mutual mutilation of individuals by satisfying the nutritional needs with the necessary substances (Haščík et al., 2014; Karpiński et al., 2014).

#### 4.3.2 Collecting pollen

Bees are the pollinators of most plants in the world. Thanks to honeybees, it is possible to produce food that meets the needs of mankind, bringing unimaginable profits to the world economy. The collection of flower pollen is carried out with the help of various collectors; verandas, bottom collectors, or cap collectors. The insects returning to the hive squeeze through the hole in the plastic plate, losing the pollen deposits that fall into the collector's tray. Each received batch of pollen should be preserved to protect it from microbial contamination. Most often this is done using dryers at a temperature of +40°C. It is worth noting that exceeding this temperature will denature proteins and the loss of many valuable nutrients contained in pollen. The water content of fresh pollen drops from 18%–25% to 4%–6%. After drying, the pollen is cleaned of light impurities in special dressers. Dried pollen placed in tightly closed vessels can be frozen at −20°C. Another way to preserve pollen is to use freeze‐drying—a process that involves placing the raw material in vacuum apparatuses at a temperature of −40°C, thanks to which they are deprived of water. It is an expensive process and is used especially in the pharmaceutical and cosmetic industries. Flower pollen grains are made of a hard shell that protects against damage and thus can be stored without restrictions, surviving for millions of years (Graça et al., 2010; Kurek‐Górecka et al., 2017).

#### 4.3.3 Pollen microbial contamination

Literature data confirm the presence of microbes in bee pollen, the source of contamination of which are pollen grains and secretion from the digestive tract of bees used to form legs (Hani et al., 2012). Microbiological analysis of fresh pollen revealed the presence of mainly gram‐positive cocci of the genus *Enterococcus* and *Micrococcus*, fermenting gram‐negative bacilli of the genus *Enterobacter*, *Serratia*, *Hafnia*, *Cedecea*, and *Erwinia* as well as nonfermenting *Pseudomonas*, *Flavobacterium*, *Acinetobacter*, *Alcaligenes*, and *Aerobacteria* from the genus *Bacillus*, and the anaerobes of the genus Clostridium. Mold fungi of the genus *Aspergillus*, *Penicillium*, *Mucor*, *Rhizopus*, *Fusarium*, *Alternaria*, *Cladosporium*, and *Trichothecium* as well as yeast fungi of the genus *Rhodotorula* and *Geotrichum* have also been found. Molds from the genera *Alternaria*, *Mucor*, and *Penicillium* have been observed in pollen from Italy, Mexico, and Slovakia (Brindza et al., 2010; Bucio Villalobos et al., 2010; Nardoni et al., 2016). For pollen from Slovakia, molds from the genera *Cladosporium, Aspergillus, Aureobasidium, Humicola, Monodictys, Paecilomyces, Rhizopus, Mortierella, Trichosporiella*, and *Harpografium* have also been identified (Brindza et al., 2010). In Brazilian pollen, molds of the genus *Aspergillus, Cladosporium, Penicillium, Alternaria*, and *Mucor* have been observed (Deveza et al., 2015). In the article of (Hani et al., 2012), a microbiological analysis of raw pollen collected from various regions of Algeria was performed. The obtained results showed the presence of aerobic bacteria in all tested samples. It was observed that the total number of coliforms increased with increasing ambient temperature and humidity, respectively. Consequently, it was concluded that the consumption of unprocessed pollen may pose a risk to human and animal health, as this product may contain many microorganisms that have a detrimental effect on health. Certain critical moments in pollen production, such as collection and manipulation by beekeepers as well as improper storage, can contribute to product spoilage. Microbiological analysis of frozen bee pollen confirmed the presence of pathogenic organisms such as in fresh pollen, while dried pollen showed the safest form of consumption of this product (Hani et al., 2012; Mauriello et al., 2017).

Other studies conducted by Kędzia and Hołderna‐Kędzia (2010) proved that the bee's intestinal flora is dominated by gram‐negative bacteria from the Enterobacteriaceae family, gram‐positive rods from the genus *Lactobacillus* and *Bacillus*. In addition, the pathogenic bacteria *Paenibacillus larvae* (Al‐Ghamdi et al., 2020) and *Paenibacillus alvei*, causing malignant rot of bee broods, may be present in the tested material (Djukic et al., 2012). On the other hand, the spores of the mold fungus *Ascosphaera Apis* cause pericephalic mycosis in bees. In order to prevent the growth of microorganisms, bees provided their product with substances that inhibit the growth of many harmful bacteria.

### 4.4 Bee bread

**FIGURE 3:**
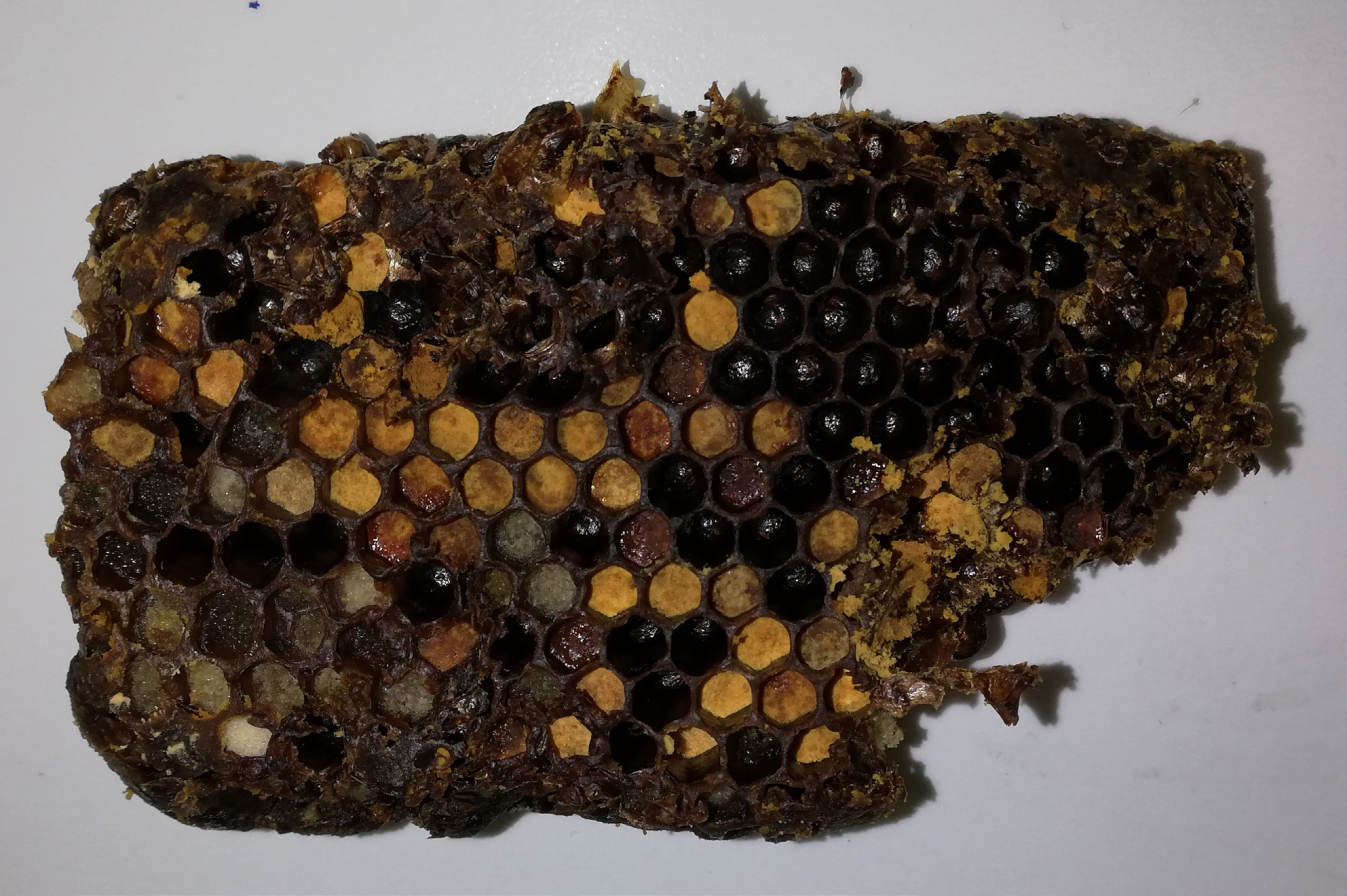
A honeycomb containing honey and bee in the cells.

In presenting the above facts about bee pollen, it is also worth quoting some important information about bee bread—a fermented final product made of a mixture of saliva, pollen, and nectar—used as food for honeybees. It is the main source of protein, lipids, vitamins, and macro‐ and micronutrients, and exhibits antioxidant and antimicrobial properties (Didaras et al., 2021). Although pollen and bee bread have the same botanical origin, the nutritional value of bee bread is higher than that of pollen (Figure 3). Bees ferment pollen probably out of the need to maintain the high quality of this product, which is not available all year round, thus increasing the bioavailability of nutrients (Kieliszek et al., 2018). The higher bioavailability of nutrients in bee bread is influenced, among other things, by lactic acid bacteria present in the digestive system of bees such as *Fructobacillus* and *Lactobacillus* (Ispirli & Dertli, 2021). Bee enzymes are involved in the maturation of bread as well as enzymes produced by its microbiome. In addition to enzymes, the bee food microbiome produces amino acids, vitamins, and antimicrobial compounds, thus increasing the nutritional value of the final product (Khalifa et al., 2021).

Bee bread contains about 20% protein, 3% lipids, 24%–35% carbohydrates, and about 3% minerals and vitamins. Fully balanced proteins containing all essential amino acids, vitamins (C, B, E, and K), pantothenic acid, dyes, and other biologically active compounds such as polyphenols (phenolic acid and flavonoids), sterols, carotenoids, and enzymes (phosphatases) are also present (Table 3). In addition, bee bread contains over 25 different micro‐ and macro‐elements such as calcium, iron, potassium, copper, zinc, selenium, and magnesium (Mărgăoan et al., 2019).

**TABLE 3:** Selected nutritional ingredients of bee bread (Baltruaityte & Rimantas Venskutonis, 2007; Čeksteryte et al., 2016; Hryniewicka et al., 2016; Markiewicz‐Zukowska et al., 2013; Zuluaga et al., 2015).

Compound	Value
DPPH (%)	89.9–94.0
ABTS (%)	73.2–92.2
Total flavonoids (mg eq‐quercitin/g)	0.14–4.5
Flavonones (mg NAE/g)	12.99
Total phenols (mg eq‐gallic acid/g)	2.5–36.5
FRAP (μmoltrolox/g)	35.0–70.1
TEAC (μmoltrolox/g)	21.0–76.3
L‐ORAC_FL_ (μmol TE/g)	3.8–11.0
H‐ORAC_FL_ (μmol TE/g)	9.34–13.0
ORAC (mg TE/g)	626.3
Total antioxidant capacity (μmol TE/g)	5.6–22.96
p‐coumaric acid (%)	0.1–0.4
Kaempferol (%)	0.08–0.4
Tocopherol (%)	0.3–0.5
Isorhamnetin (%)	0.4–0.9
Chrysin (%)	Trace
Apigenin (%)	Trace
Provitamin A (mg/100 g)	200–875
Vitamin E 9 (mg/100 g)	43.6–170.0
Coenzyme Q10 (μg/g)	8.7–14.6
Coenzyme Q10 (μg/g)	6–2000

Bee bread has been shown to have a high moisture content, ranging from 6 to 9/100 g. This is attributed to the hygroscopic properties of the pollen, which attracts water from the environment. Bee bread accumulates water from the honey, the environment, and also from the bees' saliva, which produces a sticky end product. The water content of fresh bee bread provides a favorable environment for the development of beneficial bacteria (Kieliszek et al., 2018; Mohammad et al., 2021), as well as pathogenic bacteria, fungi, and molds. This raises concerns about the risk of adverse microorganisms such as those found in pollen (*Clostridium, Bacillus*) (Lozo et al., 2015). Bee bread is more acidic than bee pollen; the pH ranges from 3.2 to 5.9. The pH value drops with increased lactic acid content, which indicates lactic acid fermentation by bacteria and yeast. It is claimed that bee bread contains all the essential amino acids that the human body cannot synthesize. Among the amino acids, lecithin and phenylalanine are predominant (Mohammad et al., 2021).

The scientific article by Didaras et al. (2020) confirmed that the protein content in bee bread is higher by about 20% compared to pollen. The total content of phenols and flavonoids increased from 1.24 to 2.40 times in pollen after the fermentation process. Among the polyphenols, the most important group of compounds present in bee bread are flavonoids. These bioactive compounds are very important for their antiallergic, anti‐inflammatory, and anticancer properties (Mărgăoan et al., 2019). In addition, they are one of the most important natural antioxidants that are present in vegetables, fruits, tea, herbs, and essential oils. Bee bread contains antioxidant compounds such as phenols and vitamin C (Sawicki et al., 2020); therefore, it has the ability to remove free radicals.

Bee bread has diverse fatty acid profiles (Čeksteryte et al., 2016; Mayda et al., 2020), which have a positive effect on bee nutrition as well as human health. Bee pollen contains the most fatty acids; polyunsaturated fatty acids (PUFA) such as omega‐3 (α‐linolenic), omega‐6 (linoleic acid), and palmitic fatty acids that the human body cannot synthesize. The fatty acid composition does not differ greatly between bee pollen and bee bread (Mohammad et al., 2021).

The chemical composition of bee bread depends on the type of plant, geographic origin, and climatic conditions. This is evidenced by the research of scientists (Kaplan et al., 2016; Simopoulos, 2004), who examined eight bee bread samples from various regions and climatic conditions in Turkey (Adana, Urfa, Mersin, Zonguldak, Edirne, Adaiyaman) and different altitudes from the sea. The research was carried out in June–October in terms of the content of proteins and lipids as well as to identify the fatty acids present. The types of flora in the study were as follows: cotton, citrus, chestnut, sunflower, and clover. In the presented research results, it was noted that clover bee bread samples had the highest protein content (22.6 and 24.2 g/100 g), while cotton was characterized by the lowest content of this component (14.8 and 15 g/100 g). Clover and citrus bee bread also had the highest fat content (10.4 and 11.5 g/100 g). In the bee bread samples obtained from the above‐mentioned plants, 37 fatty acids were identified, including 20 saturated and 17 unsaturated (including linoleic and alpha‐linolenic, from the group of essential unsaturated fatty acids).

The total unsaturated fatty acid content was higher than the sum of the saturated content identified in all samples, except for the citrus sample (Kaplan et al., 2016). Fatty acids are important for the fertility and health of honeybees (Brutscher et al., 2019). Unsaturated fatty acids also have a beneficial effect on the human body; they reduce the level of triglycerides and cholesterol in the blood (von Schacky & Harris, 2007) and have anti‐inflammatory and anticoagulant properties (Simopoulos, 2004). The aforementioned literature suggests that bee bread is a good source of polyunsaturated fatty acids (PUFA) (Bakour et al., 2019), which are crucial in human nutrition. PUFAs cannot be synthesized endogenously in the human body and must be obtained from food. The results of the experiments showed that the protein and lipid content of the bee bread depends on the pollen of the plants. The literature presented above suggests that bee bread is a good source of polyunsaturated fatty acids (PUFA) (Kaplan et al., 2016).

It is worth noting that the geographical origin of bee bread also affects the species composition of the microbial microflora, which has a direct impact on the chemical composition of this product. Scientists from the United States Department of Agriculture (Degrandi‐Hoffman et al., 2013) investigated differences in pollen‐to‐bee bread conversion by European (EHB) and African (AHB) honeybees. Bee bread produced by EHB was characterized by a higher acidity (pH 3.92) than AHB (pH 3.97) with an average pollen pH of 4.7. The lowering of the pH during the conversion of pollen to bee bread is attributed to the activity of lactic acid bacteria, which are added to the pollen from the digestive system of bees (Gilliam, 1997). However, even slight differences in pH between European (EBB) and African (ABB) bee bread suggest that the composition of microbial microflora in ABB and EBB may not be the same, especially in relation to microorganisms that synthesize acid compounds (bacteria, molds) (Degrandi‐Hoffman et al., 2013).

The protein concentration in bee bread was lower than in pollen, while the content of most amino acids was higher. One of the ways to increase the content of free amino acids in bee bread is the activity of microorganisms that produce proteolytic enzymes (Degrandi‐Hoffman et al., 2013). An alternative may be the de novo synthesis of amino acids (from primary biomolecules) by the bee bread microflora (Metges, 2000). The levels of tryptophan, proline, and cysteine were higher in bee pollen, which may be due to the use of amino acids by microorganisms (with bee bread) as a source of carbon and energy. The reduction of tryptophan content in bee bread may be related to the presence of indole bacteria (*Escherichia coli* and *Enterococcus faecalis*), which use tryptophan as an energy source, transforming it into indole. Likewise, proline concentrations were lower in EBB than in pollen and ABB. All these results could be due to the presence of microorganisms with specific amino acid degradation pathways (Degrandi‐Hoffman et al., 2013). Bee bread is an environment with high osmotic pressure (Kieliszek et al., 2018); therefore, the microorganisms present in it should be tolerant to such environmental conditions. According to (Di Martino et al., 2020), proline is one of the osmoregulatory substances used by microorganisms.

#### 4.4.1 Industrial use of bee bread

In recent years, there has been a lot of interest in bee bread. This valuable product is introduced to the market as a dietary supplement and as a functional food additive which is gaining more and more followers. In the health food market, bee bread is less available than pollen, which is much easier to extract from the hive; however, due to the stronger composition of the bread compared to pollen, it can be administered in smaller amounts or for a shorter period of time to achieve the desired health effects (Bayram et al., 2021; Didaras et al., 2021; Kieliszek et al., 2018).

The antimicrobial activity of bee bread is mainly due to polyphenols, fatty acids, and phytosterols as well as proteins and protein compounds present in the pollen from which bee bread is made (Didaras et al., 2020, 2021). The presence of many different acids in bee bread suggests that extracts from this type of bee product could be used as natural food preservatives (Bakour et al., 2022). Bee bread can also be an alternative source of nitrogen in the daily diet. A given bee product is a source of methionine or arginine, amino acids that perform a number of important functions in human metabolism. The first of them participates in DNA methylation and protein synthesis, it also acts as an antioxidant, and it eliminates excess reactive oxygen species (protection of cells against oxidative stress). Arginine, in turn, strengthens the immune system by stimulating the activity of T lymphocytes and macrophages (Bakour et al., 2022). Khalifa et al. (2020) suggest that bee bread has a hypolipidemic effect by lowering the level of cholesterol produced by lactic acid bacteria. This makes it possible to use bee bread as an additive to fermented milk products. What is more, the presence of *Lactobacillus* and other lactic acid bacteria in bee bread creates the potential for obtaining natural compounds from this kind of bee product that are used in the food industry, for example, as food preservatives. Some scientists suggest that bee bread should be used as a preservative and a dietary supplement to counteract oxidative stress and related diseases (heart diseases, cancer) (Bakour et al., 2021; Elsayed et al., 2021). Bee bread has been shown to have antimicrobial activity against *Salmonella enteriica* (Kacániová et al., 2012). It also affects the bacteria *Pseudomonas aeruginosa* (Fatrcová‐Šramková et al., 2013; Kahraman et al., 2022), which is also a safety indicator in the risk assessment of water fit for human consumption. The antimicrobial activity of bee bread against methicillin‐resistant pathogenic bacterium *Staphylococcus aureus* was also observed (Kaškonienė et al., 2020; Urcan et al., 2018), which is an indicator of a microbiological criterion for foodstuffs in the hygiene of the production process in the food industry. Therefore, it can be concluded that in the case of bee bread, its final composition is determined by the pollen fermentation process and the type of nectar.

#### 4.4.2 Microbial contamination of bee bread

The consumption of bee bread results from the ever‐growing demand for natural products that, apart from being of high nutritional value, have health‐promoting properties. One of the most important features of food consumed by humans is its safety (Kieliszek et al., 2018), therefore, with the increasing interest in bee bread, concerns about its sanitary quality are growing. This fact is related to the presence of microorganisms that can produce metabolites that are toxic to the human body, including mycotoxins (aflatoxin, ochratoxin, patulin) (Kostić et al., 2019; Mohammad et al., 2020). The presence of microorganisms in food products is not always associated with negative effects on humans, bee bread is an ideal example of this. Bee bread is an environment of a wide group of microorganisms that includes bacteria, yeast, and mold fungi, which can be characterized by outstanding biotechnological properties. Research on the microflora inhabiting the bee bread is aimed at isolating new groups of microorganisms that can be used in various industries.

During the formation and maturation of bee bread, complex biochemical changes take place as a result of the activity of enzymes and microorganisms (Khalifa et al., 2020), mainly *Lactobacilli*. As a result of the above‐mentioned changes, the digestibility and bioavailability of nutrients increase. Bee pollen and bee bread digestibility studies show differences between the values of bee bread (79.1 g digested protein/100 g total protein) and bee pollen (63.9 g digested protein/100 g total protein) (Zuluaga et al., 2015). The conversion of bee pollen to bee bread is due to the activity of lactic acid bacteria, which gain energy as a result of the transformation of sugars present in the environment into lactic acid. As with other fermented products, after the fermentation process is complete, high levels of lactic acid (3.2%, pH 4.1–4.4) and other metabolites (organic acids, diacetyl, acetaldehyde, bacteriocins) preserve the bee bread, preventing spoilage (Kieliszek et al., 2018). The most important representatives of lactic acid bacteria are *Lactobacillus* and *Bifidobacterium*, which are the main microflora living in the intestines of honeybees (Gilliam, 1997). Studies carried out in recent years have shown that metabolites produced by *Lactobacilli* contribute to the inhibition of the development of pathogenic microorganisms. The growing interest in lactic acid bacteria is due to the fact that they are on the gras list, that is, generally recognized as safe for health (Plavec & Berlec, 2020). The starters of the above‐described bacteria have found application, among others, in the dairy, fruit, and vegetable, and meat industries (Gilliam, 1997; Khalifa et al., 2020).

As a result of the isolation and identification of yeast by Gilliam (Gilliam, 1997), 113 yeast strains were obtained from pollen and bee bread, which belonged to seven genera: *Cryptococcus, Kloecker, Candida, Rhodotorula, Torulopsis, Hansenula*, and *Metschnikowia*. The genus *Torulopsis, Candida*, and *Cryptococcus* accounted for approximately 85% of the identified strains. Torulopsis magnoliae was the most common representative of yeast (43% of the strains obtained). This organism was also the most common yeast isolate from the gut of adult honey laborers in Arizona (Gilliam et al., 1977). Because *T. magnoliae* was not found in flower pollen, but first appeared in the bee bread, it was then found in all bee bread samples, which means that the organism's home is the bee's digestive tract. Overall, most pollen yeasts have not been identified in bee bread. It should be noted that the physical and chemical properties of pollen and bee bread may influence the composition of the microflora of this product (Gilliam, 1979).

A study by Nogueira et al. (2012) on bee pollen from Portugal showed a correlation between a high water activity coefficient (aw) and an increased amount of isolated microorganisms from the samples. It turned out that at pH values >4.96 and a correspondingly high water activity coefficient >0.55, increased multiplication of some microorganisms was observed.

The high osmotic pressure associated with the high sugar content and the honey layer in the bee, which reduces the access to oxygen, limits the development of some yeast strains. The yeasts isolated during the research (Gilliam, 1979) were adapted to difficult growth conditions, thanks to their outstanding properties, which include, among others, assimilation of various carbon sources, starch synthesis, gelatin liquefaction, and vitamin synthesis. In order to confirm this fact, the growth of isolated yeasts, derived from pollen and bee bread on a substrate without the addition of vitamins, was investigated. The vitamin deficiency test showed that 13% of the strains showed an increase in the absence of vitamins in the medium.

#### 4.4.3 Acquiring bee bread

The bee bread are taken out of the honeycombs by hand using a special tube with a diameter of 5 mm with sharpened edges, which is equipped with a spring piston mechanism inside. The bee bread obtained in this way is stored in airtight containers at low temperature and in a dry, dark place (Uracan et al., 2017).

### 4.5 Propolis

**FIGURE 4:**
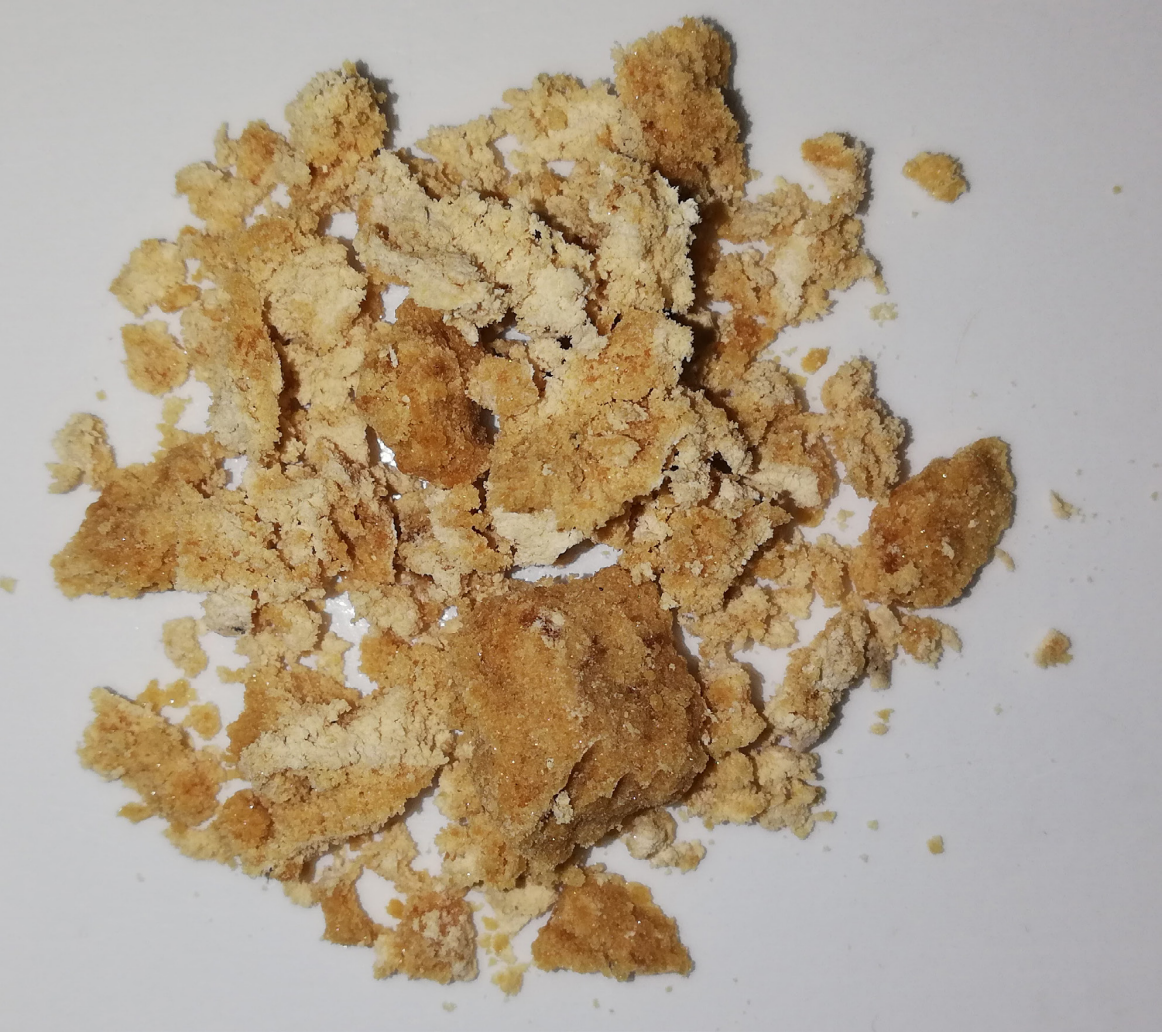
Bee wax.

Another bee product with a wide pro‐health effect is propolis (Kurek‐Górecka et al., 2020). Propolis, or bee putty, is a natural resin collected by bees from the buds of various trees, green plants, and shrubs. The word propolis is derived from Greek and refers to entering a city (Anjum et al., 2019). The bee family uses propolis putty as a material to secure the nest, creating a protective barrier inside the hive (Tautz & Steen, 2018), including against pathogenic microflora as well as for mummification of intruders, thus preventing their decay. The bees collect small pieces of resinous substances from the cracks of the tree bark and buds with the help of mandibles, moistening them with saliva and forming a lump. Then, they put it in baskets on the legs and transport it to the hive. The resinous substance collected from the buds and other parts of the plants is mixed with the enzymes of the salivary glands and beeswax (Figure 4) to form propolis. This raw material is most abundant in the hive in late summer and autumn when the bees begin to seal the nest before winter (Przybyłek & Karpiński, 2019).

Propolis has a very variable and complex chemical composition. It depends on the region and climate in which it is produced, and on the species of plants used to obtain it. However, chemically differentiated bee putty has similar anti‐inflammatory, antiviral, antioxidant, antibacterial, and antifungal effects (Béji‐Srairi et al., 2020; Zulhendri et al., 2021). The color of propolis ranges from yellow, through orange, green, and even dark brown—it depends, among other things, on the source of its origin. At low temperatures, propolis hardens and becomes brittle, while at higher temperatures, it is soft and very sticky (Ceylan, 2021; Kuropatnicki et al., 2013). It has an aromatic smell, sometimes bitter and pungent. One bee colony can produce from 50 to 150 g of propolis per year (Mountford‐McAuley et al., 2021), and it is most often obtained by scraping from different parts of the hive (Stawiarz & Dyduch, 2014).

The main chemical compounds found in propolis are resins (approx. 40%–50%), waxes (approx. 20%–30%), pollen (approx. 5%), polyphenols (approx. 14%–16%), terpenes (approx. 8–12.5), and essential and aromatic oils (approx. 10%). In smaller amounts, less than 1% are microelements such as iron, copper, phosphorus, manganese, zinc, calcium, cobalt, magnesium, and selenium. A small amount of vitamins, such as vitamins B1, B2, B6, C, and E, have been identified in the bee putty (Mohdaly et al., 2015; Pobiega et al., 2017; Szeleszczuk & Zielińska‐Pisklak, 2013).

Propolis is also a source of carbohydrates, enzymes, sterols, and fatty acids. Flavonoids, aromatic esters, and terpenes are considered to be the most important components with the greatest biological importance. Flavonoids are natural, water‐soluble chemical compounds that fight free radicals, have antioxidant properties (Huang et al., 2014), improve the elasticity of blood vessels, delay the aging process, inhibit the accumulation of cholesterol, protect the circulatory system, and lower blood pressure. The flavonoids found in propolis are mainly chrysin, apigenin, quercetin, galangin, pinocembrin, and pinostrobin (Kurek‐Górecka et al., 2020; Woźniak et al., 2018). Aromatic esters included in propolis show bactericidal properties. These include, among others, benzyl, pentylene, and phenethyl esters as well as caffeic and cinnamic acid (Aliboni et al., 2011). Terpenes are substances that allow plants to protect themselves from pests, including fungi and bacteria. Due to their properties, they are used in the food and pharmaceutical industries as well as in the production of cosmetics. These include, among others, geraniol, nerol, and farnesol (Przybyłek & Karpiński, 2019).

In Polish propolis, on the basis of numerous studies, it has been confirmed that the content of flavonoid compounds is about 6.2%–18.8%, the majority of which are pinocembrin (about 2.2%) and chrysin (about 2.1%). Terpenes and polyphenolic compounds correspond to a specific smell and contribute to the biological activity of propolis (Anjum et al., 2019; Przybyłek & Karpiński, 2019).

#### 4.5.1 Obtaining propolis

Beekeepers obtain propolis by scraping it from the walls of the hive, frames as well as trap bars or covers. The use of hives in the Apipol system gives much better results in obtaining propolis. This system uses plastic inserts with trapezoidal slots cut into the cover. It is the newest and most hygienic method of obtaining pure bee putty. The sticky inserts with propolis are placed in a refrigerating device because the cold putty comes off more easily from the smooth surface of the material.

Propolis is harvested in both temperate and tropical zones (Al‐Waili, 2018). In Central Europe, including Poland, bees collect exudate from deciduous tree buds—poplar, alder, birch, willow, oak, ash, chestnut, and from the bark of conifers—spruce, fir, and pine. Propolis produced in other European countries such as Albania, Bulgaria, Hungary, and England, and in many regions of the temperate zone, including the USA, West Asia, and North Africa, has a similar origin. However, in countries with a tropical climate, the product is made from exotic plants such as Acacia (North Africa), Xanthorrhoea (Austria), Baccharis, Araucaria, Eucalyptus (Brazil), Clusia (Central America), and Plumeria (Hawaii).

#### 4.5.2 Properties of propolis and its use

In the food sector, propolis is added to wine, cookies, chewing gum, and candies. In the cosmetics industry, it is used as an ingredient in body lotions, shampoos, and toothpaste, and as an ingredient in antiseptic mixtures. Propolis finds clinical application in the treatment of skin diseases, including in the treatment of fungal stomatitis *(*Anjum et al., 2019 *)*. Propolis can be found in dentifrices, lozenges, mouth rinses, creams, gels, and cough syrups.

In the food industry, propolis is used as a preservative in processed and unprocessed meat (Pobiega et al., 2019). In marinating processes, it creates a natural, foil‐like coating on the surface of meat products, which prevents the activity of microorganisms. The use of ethanol propolis extract for the treatment of beef‐cured meats allows for extending the shelf life without showing any negative impact on the quality characteristics of the meat. On the other hand, the use of propolis as a natural food preservative contributed to the neutralization of pathogenic bacteria (SI Al‐Adham et al., 2022). It has also been proven that propolis resin can be used as a preservative for unpasteurized fruit juices (Liao et al., 2021), of which the main cause of spoilage is yeast. Due to its characteristic numerous health‐promoting properties, propolis is used in various industrial fields as an additive to food, including dietary supplements, and as an additive to medicines and cosmetics, gaining an ever‐increasing number of interested people (Yucel et al., 2017). Propolis can be used as a component of coatings used in the food industry. A propolis coating used on the surface of fruit or vegetables can help reduce the use of synthetic packaging. In addition, such a coating modifies the inner atmosphere of the fruit and vegetables, retaining the texture for a longer period of time (Irigoiti et al., 2021; Tavares et al., 2022).

The antibacterial effect of propolis results from its direct action on microorganisms (Gavanji & Larki, 2017). As a result of the analysis of the mechanism of propolis action, it was observed that the biological activity of this bee product is higher in relation to gram‐positive than gram‐negative bacteria. This is associated with the construction of the wall–membrane complex of gram‐negative bacteria and the production of hydrolytic enzymes that break down the active substances of propolis. In addition, propolis resin also has a bactericidal effect and it stops the division of the bacterial cell by inhibiting protein synthesis and destroys parts of the structure of the bacterial cell—the cell wall and cytoplasm (Anjum et al., 2019; Przybyłek & Karpiński, 2019).

It is believed that the flavonoids and esters of phenolic acids are responsible for the antimicrobial properties of propolis. One of the richest sources of compounds is the ethanol extract of propolis (EEP). Pinocembrin and apigenin are among the basic representatives of the flavonoids in propolis. In another study on the antibacterial effect of propolis (Anjum et al., 2019), the effect of pinocembrin on *Streptococcus mutant, Staphylococcus aureus, Listeria monocytogenes, Pseudomonas aeruginosa*, and *Klebsiella pneumoniae* and the effect of apigenin were found on gram‐negative bacteria such as mirabilis, *Pseudomonas aeruginosa*, *Klebsiella pneumoniae*, and *Enterobacter aerogenes*. The properties of propolis have also been demonstrated against other aerobic bacteria such as *Bacillus cereus, Bacillus subtilis, Enterococcus faecalis, and Micrococcus luteus*, and against anaerobic bacteria such as *Escherichia coli* (Anjum et al., 2019; Ejsmont et al., 2015).

The antifungal activity of propolis is related to the synergistic action of all the components present in its composition. The most frequently mentioned group of compounds that are responsible for this activity and which can be prescribed are phenolic compounds and terpenes. It has been proved (Pobiega et al., 2017) that propolis components inhibit mycelium growth by affecting the cellular respiration of the pathogen's hyphae, which leads to energy loss, followed by rupture of the cell membrane and cell death. Research on propolis confirms (Petruzzi et al., 2020) its activity against various fungi. Flavonoids such as pinocembrin, galangin, and chrysin, which have been identified in propolis resin, are characterized by very high antifungal activity (Cibanal et al., 2020). Galangin and pinocembrin inhibit the growth of C*andida albicans, Candida tropicalis, Saccharomyces cerevisiae, and Candida neoformans*, including galangin, additionally showing the ability to inhibit the development of *Trichophyton mentagrophytes* and *Trichophyton rubrum*. Chrysin, on the other hand, has little effect on growth inhibition of most fungi of the genus *Candida, Aspergillus, Penicillium*, and *Trichoderma virens* (Pobiega et al., 2017).

The bee product propolis also shows antiviral activity (Siheri et al., 2017). *Responsibility for this is attributed to the com*ponents present in the propolis resin such as flavonoids, caffeic acid, and esters of aromatic acids. It is likely that these compounds work by inhibiting the entry of the virus into other cells and destroying the outer shell of the virus. Antiviral properties have been proven against the herpes simplex virus HSV‐1 and HSV‐2 (Demir et al., 2020). Propolis contributes to faster wound healing and resolution of local ailments (Pobiega et al., 2017). Nakamura et al. (2013) proved the beneficial effect of propolis on the liver in rats infected with viral hepatitis. The researchers gave the rats an agent that had a proven harmful effect on hepatocytes, and then divided the animals into three groups: the first group was given ethanol extract from propolis, the second group was given vitamin E, and the third group was the control group. In rats that received propolis extract, no cholestasis or damage to liver cells was noted, in the second group there was no cell damage, but cholestasis was not prevented, while in the control group, there was damage to hepatocytes, cholestasis, and high cholesterol and triglyceride concentrations. Propolis also has a soothing effect, which is why it has been used in the treatment of dermatological diseases (inflammation of the sweat glands and hair follicles). Propolis has also been used in wounds caused by thermal damage to the skin and postoperative wounds (Kurek‐Górecka et al., 2020; Rojczyk et al., 2020). Its anti‐inflammatory effect makes it effective against diseases of the throat and larynx as well as inflammations and infections of the oral cavity such as aphthae or thrush, and it is also used in dentistry (Szeleszczuk & Zielińska‐Pisklak, 2013; Zulhendri et al., 2021).

The antioxidant mechanism of propolis is closely related to the action of phenolic compounds that give back hydrogen ions to free radicals in order to protect cells against oxidation, and also protect against oxidation reactions of stored food. The components of propolis such as phenolic acids, galangin, and pinocembrin, which are part of the flavonoids, have the ability to remove radicals and protect cells against aging (Anjum et al., 2019; Spanidi et al., 2021).

### 4.6 Royal jelly

Royal jelly is a natural substance with a creamy texture, providing food for larvae in the first days of their life (Neacsu et al., 2022) and for bee queens throughout the entire larval period. Royal jelly is a secretion from the pharyngeal and mandible glands of worker bees, the so‐called nurses. It takes various colors from yellow (Ramadan & Al‐Ghamdi, 2012) to cream, to dark yellow (Kausar & More, 2019). The acidity of the milk ranges from pH 4.1 to pH 4.8. The taste is tart, sour, and slightly bitter (Sidor et al., 2021).

The main ingredient in royal jelly is water (approximately 50%–60%). Large groups of chemical compounds form proteins (about 17%), carbohydrates (15%)—mainly glucose, fructose, and sucrose (Xue et al., 2017), and lipids (about 3%–4%), which include, among others, waxes, phospholipids, sterols, glycerol, and fatty acids (Isidorov et al., 2012). The composition of fresh royal jelly also contains mineral compounds (about 1.5%) and vitamins (A, E, C, and the B group), nucleotides, peptides, and polyphenols (Collazo et al., 2021), with the highest concentration of phenolic acids, followed by flavones and flavonols (Peršurić & Pavelić, 2021).

In royal jelly, apart from amino acids such as albumin and globulin, there are also biologically active substances; enzymes and hormones, including the neurohormone—acetylcholine (Kamyab et al., 2019). Acetylcholine acts as a neurotransmitter and regulates the work of endocrine glands by synthesizing hormones into the bloodstream. The gamma globulin contained in the milk is responsible for regulating immune processes, giving organisms more opportunities to fight infections (Plavec & Berlec, 2020). The peptides present in the composition of the milk exhibit immunological and hypoglycemic effects by lowering blood sugar levels (Yucel et al., 2017).

The milk contains enzymes, including hydrolytic enzymes from the group of acidic glycosidases from the bees' glands (Sidor et al., 2021). The increased activity of enzymes in the milk is due to their accumulation as a result of frequent food supply to the larvae, and the nutritional components of the milk are modified by glycosidases. The acidic environment of the milk promotes the activity of enzymes (Ramadan & Al‐Ghamdi, 2012). The peptides present in the composition of the milk exhibit immunological and hypoglycemic effects by lowering blood sugar levels.

#### 4.6.1 Properties of royal jelly and its use

Royal jelly can be used as a dietary and medicinal product (Strant et al., 2019). Due to the presence of many nutrients and biostimulants (e.g., 10‐hydroxy‐2‐decenoic acid), it helps to supplement the deficiencies in the daily diet. This bee product, thanks to its nutritional properties, perfectly allows you to make up for energy losses and body weight losses in states of malnutrition. It is worth noting that the highest consumption of royal jelly occurs in Japan (approx. 300 tons per year) (Haydak, 2015; Van Toor, 2006). The largest consumers of royal jelly after Japan are Europe and North America. In some European countries, royal jelly is added to yogurts (Kavas, 2022), while in Asia it is used in various beverages (Moriyama et al., 2017). In addition, royal jelly can also be found in jellies made of honey, in sugar syrups, jam, and pectins (Bąk & Wilde, 2002). Currently, scientific work is being carried out on the production of new functional foods based on royal jelly, its properties, and its pharmacological effects on the human body (Collazo et al., 2021). Recent studies have shown that royal jelly can be used as an efficient food for patients with type 2 diabetes. This is related to the desired effect on glucose and apolipoprotein AI, which attenuates cardiovascular attack in affected patients (Khazaei et al., 2017).

Not only enzymes, lipids, and proteins, including peptides, but also nucleotides present in royal jelly are responsible for the healing effect of royal jelly. Nucleotides are characterized by immunostimulating properties, increasing mental and physical immunity, positively affecting the liver and lipid metabolism as well as the digestive system, and renewing the intestinal tissue (Kędzia et al., 2019).

Royal jelly has been shown to have antimicrobial properties (Fratini et al., 2016). The royalysin protein and certain fatty acids (3‐hydroxydodecanoic, 11‐oxododecanoic, 11‐S‐hydroxydodecanoic) are responsible for this positive feature. Royal jelly inhibits the growth of gram‐positive bacteria (*Bacillus subtilis, Staphylococcus aureus*) and gram‐negative bacteria (*Escherichia coli, Pseudomonas aeruginosa, Klebsiella pneumoniae*). It is also active against mold (*Aspergillus fumigatus, Aspergillus niger*) and yeast (*Candida albicans)*. Moreover, royalysin has been shown to be particularly active against *Paenibacillus larvae* (Ejsmont et al., 2015).

Royal jelly is used in various forms: alone or in combination with honey and other bee products, in the form of tablets as a dietary supplement. Due to its rich composition and unique properties, it is used not only in the food industry but also in the cosmetics industry (Chander & Lovleen, 2021).

#### 4.6.2 Obtaining royal jelly

Royal jelly is an unstable product and loses its biological activity due to the decomposition of its ingredients. It is used by bees to raise queen bees. Royal jelly is obtained by collecting it from 3‐day‐old queen cells when you can get the most—on average 23 mg of royal jelly. For this purpose, special devices are used, operating on the principle of sucking in the raw material. After harvesting, the raw material is stored in a hermetically sealed and dry container, preferably at low temperature, as it easily loses its nutritional properties (Saricaoglu et al., 2019). Royal jelly retains its properties for several days at room temperature. In refrigeration conditions at a temperature of 0–5°C, it can be stored for up to several months. Royal jelly can be preserved by combining it with honey and alcohol (Graça et al., 2010). The mixtures created in this way allow the finished product to be stored in an airtight vessel and in a dark place for a period of a year. The best method of preserving the milk is freeze‐drying (vacuum drying in a frozen state), which allows for long‐term storage at 0–15°C without loss of quality. Freezing prevents the breakdown of biologically active proteins.

## 5 APPLICATION OF BEE PRODUCTS

The protection of modern beekeeping and the regeneration of natural beekeeping methods indicate the great importance of bees not only for nature but also for humans (Kieliszek et al., 2018). Nowadays, there is an increased interest in more unconventional methods of treatment using biologically active substances that occur naturally in the environment (Stawiarz & Dyduch, 2014).

One branch of alternative medicine that uses bee products to treat diseases is apitherapy. Being one of the practices of complementary medicine, apitherapy involves the use of bees and bee products as adjuvants and supplements in the treatment of certain diseases (Kieliszek et al., 2018). First of all, apitherapy is practiced to strengthen the immune system and treat certain diseases of the respiratory tract and digestive system. Increasingly, products of natural origin are becoming the subject of scientific research. These include bee honey, pollen, propolis, and bee bread. The use of these products is recommended for people of all ages, except for people allergic to bee products and infants under the age of one. The most popular of the above‐mentioned products is bee honey, which is an easily digestible food and contains a lot of energy and building components. It is precisely due to the rich chemical composition that honey shows such as good healing properties and high nutritional value (Hadagali & Chua, 2014). Due to the high content of glucose and fructose, honey, in just a short time, contributes to the reduction of tiredness and fatigue as well as to the rapid renewal of energy in the body (Ali et al., 2021). Years ago, honey was used in the treatment of gastric and duodenal ulcers, burns, and skin diseases (Mandal & Mandal, 2011), as well as in the treatment of liver and kidney problems. It is also recommended for consumption in the presence of cardiac diseases because it improves circulation and blood pressure (Stawiarz & Dyduch, 2014).

The data presented by Hołderna‐Kędzia and Kędzia (2006) show that honey administered orally shows antioxidant properties that significantly reduce the level of superoxide free radicals in the human body, thus protecting it against the onset of serious diseases. One must also not forget about the antibacterial, bactericidal, and bacteriostatic properties of honey. It should be remembered that the nutritional properties are characteristic of ripe honey, which is consumed raw, because bringing it to a temperature above 40°C results in the loss of these characteristic features (Stawiarz & Dyduch, 2014).

The prospect of using bee products such as honey, propolis, and pollen in oncology is extremely important, namely, in postoperative aid and the treatment of skin and mucous membranes damaged by irradiation of neoplasms. Unfortunately, there are still few attempts to use bee products in supporting cancer treatment (Kędzia & Hołderna‐Kędzia, 2015; Premratanachai & Chanchao, 2014). The use of diluted honey on wounds resulting from the removal of a tumor from the vulva inactivated pathogenic bacteria (including *Staphylococcus aureus and Escherichia coli*), and healed the wound completely after 6–8 weeks (Cavanagh et al., 1970). Honey has also been used in the treatment of ulcers, neoplasms (honey paste, honey bandages), damage to the skin after radiation therapy (honey dressings), in preparations for the treatment of tumors on internal organs (kidneys, stomach, colon, larynx), or to increase the selenium level in the body of smokers. Bee products such as honey and propolis in the form of ointments, paste, lotions, water and alcohol extracts, and spray preparations have been used in the treatment of skin defects caused by X‐rays and ionizing radiation. For good reason, pollen can also be used in oncology. Pollen deposition in a dose of 60 g a day to people before and after radiation therapy increased the number of erythrocytes, blood hemoglobin, and vitamin E levels, and decreased cholesterol levels. The studies conducted prove the protective effect of flower pollen on the body and the alleviation of the effects of radiation sickness (Kędzia & Hołderna‐Kędzia, 2015).

The widest range of application in both apitherapy and in oncology is demonstrated by honey, which is best known in research and experiments (Kędzia & Hołderna‐Kędzia, 2015). Increasingly, scientists are focusing on the health‐promoting effects of other bee products of plant origin. The most popular of these are flower pollen and its fixed form—bee bread.

The positive effect of bee bread on the nervous system has been proven. As a result of analyses carried out on Lithuanian bee bread, two substances were isolated—kaempferol and chrysin. Kaempferol, known for its wide range of health‐promoting properties, has a proven protective effect on the nervous system. In contrast, chrysin, in addition to its neuroprotective properties, also has anticonvulsant and anxiolytic effects (Khalifa et al., 2020).

The high content of bioactive ingredients found in bee bread enables it to exhibit outstanding health‐promoting properties. The use of bee bread as a dietary supplement in a dose of 20–40 g a day for an adult man strengthens the immune system of the body, reduces allergic reactions, and supports drug treatment. The systematic consumption of bee bread regulates blood cholesterol levels, reduces the total lipid content (antiatherosclerotic effect), and is also beneficial for the functioning of the circulatory system (heart function). People who added bee bread to their food ration noticed an improvement in concentration and memory and observe the antiaging effect (due to the presence of antioxidant compounds and regeneration of the body's cells). Bee bread is widely used in cleansing the liver (it has a protective and detoxifying effect), in the treatment of intestinal diseases, anemia, and diabetes, and also in regulating the functioning of the digestive system (Kieliszek et al., 2018). Current scientific evidence has revealed the different biological properties of bee bread, namely, its anticancer, antimicrobial, and antioxidant activity.

In a study by Portuguese scientists (Sobral et al., 2017), samples of bee bread were found to be toxic to four human cancer cells. At the tested concentrations, bee bread samples (up to 400 μg/mL) inhibited less than 50% of tumor cell growth, while none of the bee bread samples showed toxicity to healthy cells. Bee bread's anticancer activity is related to the presence of phenolic compounds. In the course of their study, 32 antioxidant compounds were identified in six samples of bees collected from various apiaries near Bragança (the northeastern part of Portugal), including mainly quercetin, kaempferol, myricetin, and isorhamnetin.

Bee products in the food industry can find wide application due to their health‐promoting effect. This constantly developing industry influences the design of new food products that may interest consumers. Building information bases and, moreover, increasing the competitiveness of the economy, including the food economy, on an international, national, and regional scale, affects the increasing importance of the quality of manufactured products. The effects of implementing innovative technological solutions should bring tangible results for the entire food industry. The implemented changes may relate to food processing technology to improve its safety, for the convenience of its use or taste, and they may also be related to a change in the distribution system, packaging, or forms of promotion. Appropriately recommended bee products and marketing strategies should help to increase society's knowledge of bee products and their health‐promoting properties. Moreover, the possibility of emphasizing the cultural uniqueness of different bee products, and their distinctiveness and originality may lead to increased interest in these products.

## 6 PERSPECTIVE BEE PRODUCTS IN THE FOOD INDUSTRY

The awareness of the public regarding the influence of diet on health is constantly increasing, and this, in turn, is causing specific changes in shaping the entire food industry. Producers have to react and respond to the needs and requirements of potential consumers, as it is they who drive the food market. Thus, they are introducing more and more products of natural origin, from organic farming (bioproducts, food with good composition, and a positive effect on the functioning of the human body). More and more studies define exactly the conditions and standards that should be met with regard to these products so that they can be used and recognized as pro‐health. It is known that an important aspect is not only the geographical origin of bee products but also the quality of a given environment, its industrialization, and thus also pollution with various substances. In addition, it is important to maintain appropriate parameters at each stage of the technological process, with particular emphasis on thorough maintenance (Estevinho et al., 2011). All this guarantees a high‐quality end product that is not only safe to eat but also represents food with special nutritional properties. This, in turn, places, for example, bee bread and pollen in the area of products that deserve special interest from every consumer (Machado De‐Melo et al., 2018). The content of a large number of free amino acids, various vitamins, and minerals guarantees not only supplementation but also enrichment of the diet with substances that are not supplied in the right amount with everyday food. Thus, it becomes an extremely interesting product, which may result in enormous popularity and widespread use in the near future. All this may also be largely based on the appropriate marketing and advertising of bee products (including bee bread as a food product, of natural origin and at the same time bringing many benefits) (Belhadj et al., 2014; Machado De‐Melo et al., 2018). Appropriate conditions have been defined for bee pollen, which should be ensured for these products in order to maintain their appropriate quality, freshness, and suitability for consumption during the specified storage period. This is an important aspect from the perspective of food safety, as it is not only the production stages that pose a potential threat of various types of contamination. Further product handling, such as transport or storage, may lower the final product quality (Akhmetova et al., 2012; Estevinho et al., 2011). High nutritional quality, including pollen and bee bread, becomes a secondary aspect if they are not safe to eat. The legal standards for the marketing of apiculture products other than honey are not sufficiently defined and, until 2012, the only countries that legally set the quality standards of pollen placed on the market were Argentina, Sweden, and Portugal (Estevinho et al., 2011). There are also other standards in the EU regarding the quality of honey, but there is still no regulation on the quality and technological conditions of bee bread placed on the market. The fact is that in order for these types of products to be considered natural dietary supplements or to be used in apitherapy, their quality must be rigorously and constantly monitored (Estevinho et al., 2011). The trend of caring for a diet and consuming less processed food has not changed for several years. Consumers more and more often analyze the composition of products before buying them, which then influences their nutritional choices. Each potential consumer should have the right to decide on the purchase of a specific product based on various characteristics, that is, price, composition, quality, impact on health, appearance (color, texture), taste preferences, or product origin. All the information that can be placed on the label such as place of production, product weight, ingredients, allergens, etc., should be included on the label, thereby allowing consumers to verify and make informed choices (Nogueira et al., 2012). The obligation to place this type of information on the label should be regulated and controlled legally in every country in which bee products, including bee bread, are released for retail sale.

## 7 CONCLUSION

Increased interest in biologically active natural products is related to their pro‐health and healing properties, but also to the increased environmental awareness of consumers and the growing fashion for a healthy lifestyle. The dynamically developing market of food products makes the good quality of products, especially natural ones, a priority demand factor. Getting to know some of the positive features of bee products arouses even greater interest in scientists who go deeper in their research—examining the chemical and microbiological composition. The need for education and awareness raising should cover the entire area of the developing economy, but also its society as potential buyers. In order to distinguish themselves from ecological technologies, food producers should invest in education and development because, without innovation in the field of food technology and biotechnology, it will be difficult to maintain competitiveness and, consequently, consumer interest in bee products.

## AUTHOR CONTRIBUTIONS

**Marek Kieliszek:** Conceptualization (lead); data curation (lead); project administration (lead); writing – original draft (lead); writing – review and editing (lead). **Kamil Piwowarek:** Conceptualization (equal); writing – original draft (equal). **Anna M. Kot:** Data curation (equal). **Marta Wojtczuk:** Data curation (equal); writing – original draft (equal). **Marek Roszko:** Data curation (equal). **Marcin Bryła:** Data curation (equal); writing – original draft (supporting). **Anka Trajkovska Petkoska:** Writing – original draft (supporting).

## ACKNOWLEDGMENTS

The authors would like to acknowledge the support of the Warsaw University of Life Sciences ‐ SGGW, Poland.

## CONFLICT OF INTEREST STATEMENT

The authors declare no competing interests.

## Data Availability

Data sharing is not applicable and no new data are generated and this review article describes entirely the published literature.
